# Shared and Distinct Phenotypes and Functions of Human CD161++ Vα7.2+ T Cell Subsets

**DOI:** 10.3389/fimmu.2017.01031

**Published:** 2017-08-30

**Authors:** Ayako Kurioka, Aminu S. Jahun, Rachel F. Hannaway, Lucy J. Walker, Joannah R. Fergusson, Eva Sverremark-Ekström, Alexandra J. Corbett, James E. Ussher, Christian B. Willberg, Paul Klenerman

**Affiliations:** ^1^Peter Medawar Building for Pathogen Research, University of Oxford, Oxford, United Kingdom; ^2^Department of Microbiology and Immunology, University of Otago, Dunedin, New Zealand; ^3^Institute of Cellular Medicine, Newcastle University, Newcastle upon Tyne, United Kingdom; ^4^Department of Molecular Biosciences, The Wenner-Gren Institute, Stockholm University, Stockholm, Sweden; ^5^Department of Microbiology and Immunology, Peter Doherty Institute for Infection and Immunity, University of Melbourne, Melbourne, VIC, Australia; ^6^National Institute for Health Research Biomedical Research Centre, University of Oxford, Oxford, United Kingdom

**Keywords:** mucosal-associated invariant T cells, innate-like T cells, MHC class I-related protein 1-tetramer, MHC class I-related protein 1, subsets, transcription factors, CD8 coreceptor

## Abstract

Human mucosal-associated invariant T (MAIT) cells are an important T cell subset that are enriched in tissues and possess potent effector functions. Typically such cells are marked by their expression of Vα7.2-Jα33/Jα20/Jα12 T cell receptors, and functionally they are major histocompatibility complex class I-related protein 1 (MR1)-restricted, responding to bacterially derived riboflavin synthesis intermediates. MAIT cells are contained within the CD161++ Vα7.2+ T cell population, the majority of which express the CD8 receptor (CD8+), while a smaller fraction expresses neither CD8 or CD4 coreceptor (double negative; DN) and a further minority are CD4+. Whether these cells have distinct homing patterns, phenotype and functions have not been examined in detail. We used a combination of phenotypic staining and functional assays to address the similarities and differences between these CD161++ Vα7.2+ T cell subsets. We find that most features are shared between CD8+ and DN CD161++ Vα7.2+ T cells, with a small but detectable role evident for CD8 binding in tuning functional responsiveness. By contrast, the CD4+ CD161++ Vα7.2+ T cell population, although showing MR1-dependent responsiveness to bacterial stimuli, display reduced T helper 1 effector functions, including cytolytic machinery, while retaining the capacity to secrete interleukin-4 (IL-4) and IL-13. This was consistent with underlying changes in transcription factor (TF) expression. Although we found that only a proportion of CD4+ CD161++ Vα7.2+ T cells stained for the MR1-tetramer, explaining some of the heterogeneity of CD4+ CD161++ Vα7.2+ T cells, these differences in TF expression were shared with CD4+ CD161++ MR1-tetramer+ cells. These data reveal the functional diversity of human CD161++ Vα7.2+ T cells and indicate potentially distinct roles for the different subsets *in vivo*.

## Introduction

Mucosal-associated invariant T (MAIT) cells are a population of innate T lymphocytes expressing the semi-invariant T cell receptor (TCR), classically consisting of Vα7.2-Jα33/Jα12/Jα20 in humans, and Vα19-Jα33 in mice, which are paired with a restricted Vβ repertoire (1–4). This TCR allows them to recognize riboflavin metabolite-based antigens and folate derivatives presented by the highly conserved, monomorphic major histocompatibility complex (MHC) class I-related protein 1 (MR1) (5–7).

The identification of MAIT cells using MR1-tetramers has clearly demonstrated that MAIT cells can be divided into CD8+, CD4+, and CD8− CD4− (double negative; DN) subsets (4, 5, 8). The frequency of each subset and their distribution varies between mammalian species and is also influenced by age and tissue location. In ruminants and Vα19-Jα33 TCR-transgenic mice, the majority of the MAIT cells are DN (9, 10), whereas within humans they are predominantly CD8+ (4, 11, 12). Furthermore, while more than half of these CD8+ MAIT cells express the CD8αα homodimer in adults, MAIT cells in second trimester fetal thymi express the CD8αβ heterodimer, with CD8αα expression associated with the acquisition of a memory phenotype (12, 13). The frequency of these CD8+ MAIT cells in the periphery falls with age (14–16). Furthermore, a recent study using MR1-tetramers in wild-type mice has demonstrated that, similar to humans, certain laboratory strains of mice have a larger fraction of MAIT cells that are CD8+ (8). Furthermore, the distribution of murine MAIT cell subsets differs between tissues, with an enrichment of CD4+ MAIT cells in lymph nodes (8). Altogether, these reports highlight the heterogeneity of the frequency and distribution of MAIT cell subsets. However, the functional relationship between the different subsets, especially in humans, is poorly understood.

Mucosal-associated invariant T cells share many developmental and functional features with invariant natural killer (NK) T (iNKT) cells. Similar to MAIT cells, CD1d-restricted iNKT cells consist of CD4+ and CD4− subsets (17–19), where CD4− iNKT cells secrete T helper 1 (Th1) cytokines, as well as interleukin-17 (IL-17), while CD4+ iNKT cells are the dominant producers of Th2 and immunoregulatory cytokines, such as interleukin-4 (IL-4), IL-10, and IL-13 (18, 20, 21). The balance between these subsets is thought to be key in determining the protective or pathological role of iNKT cells in disease (22). For example, while CD4+ iNKT cells protected non-obese diabetic mice from developing type 1 diabetes, CD4− iNKT cells secreting IL-17 exacerbated the disease (22). Furthermore, the cytolytic and Th1 cytokine-producing CD4− iNKT cells are the main effector population in tumor rejection (23) and control of microbial infections (24, 25). Most of the previous MAIT cell studies have focused on the dominant population of MAIT cells in mice (DN) and humans (CD8+). Therefore, whether the different subsets have the potential to play a different function in disease remains unknown. Furthermore, whether differential expression of transcription factors (TFs) may account for the differences in MAIT cell subsets has not been fully investigated.

In humans, MAIT cells can be identified as cells expressing the C-type lectin-like receptor CD161 at a high level together with the Vα7.2 TCRα chain (4, 11, 26). These cells have previously been shown to overlap with cells stained with the MR1-tetramer, particularly in the CD8+ and DN subsets (4, 27). High expression of CD161 is a feature of innate-like T cells that have the ability to respond to innate cytokines and share a transcriptional signature, regardless of the specificity of their TCR (28), and CD161++ Vα7.2+ T cells—the majority of which are MAIT cells—are contained within this family of T cells. In this study, we have examined the frequency of CD161++ Vα7.2+ T cell subsets in human peripheral blood, liver, and bone marrow (BM), and performed a detailed analysis of the overlapping and distinct phenotypic and functional features of each.

## Materials and Methods

### Blood and Tissue Samples

Whole blood was obtained from leukocyte cones (NHS Blood and Transplant); 2-year-old donors (obtained from a cohort of Swedish infants) (29); or umbilical cord blood samples (Stem Cell Services, NHS Blood and Transplant); or healthy laboratory volunteers. Intrahepatic lymphocytes were collected from donors after portal flush using cold preservation solution following removal of the right lobe of the donor’s livers (non-pathological liver grafts preceding liver transplantation; Duke-NUS Graduate Medical School, Singapore) as previously described (30). The BM samples were obtained from routine hip joint operations (Newcastle University). Samples were filtered (40 µm), washed with phosphate buffered saline (PBS) and homogenized. Mononuclear cells from the above blood and tissues were isolated by standard density gradient centrifugation (Lymphoprep™ Axis Shield Diagnostics). Blood and tissue samples were cryopreserved and thawed before use. Adult and cord blood samples were collected after ethical approval by the Central Office for Research Ethics Committees (COREC, local research ethics committee Oxford), reference number COREC 04.OXA.010. Liver samples were collected after ethic approval of the Asian American Liver Center Ethic committee (Glean Eagle Hospital, Singapore), reference number PIEC/2012/037. For samples used for genomic DNA (gDNA) analysis, samples were collected after ethical approval by the University of Otago Human Ethics Committee (Health), reference number H14/046. All patients from the studies above provided their informed written consent. The collection of blood samples for the 2-year-old study cohort was approved by the Human Ethics Committee at Huddinge University Hospital, Stockholm, reference code 75/97, 331/02, and the parents provided their informed verbal consent. No written documentation of the participants informed approval was required, which was agreed to by the Human Ethics Committee and was according to the regulations at the time of the initiation of the study.

### Flow Cytometry

**Figure 1:**
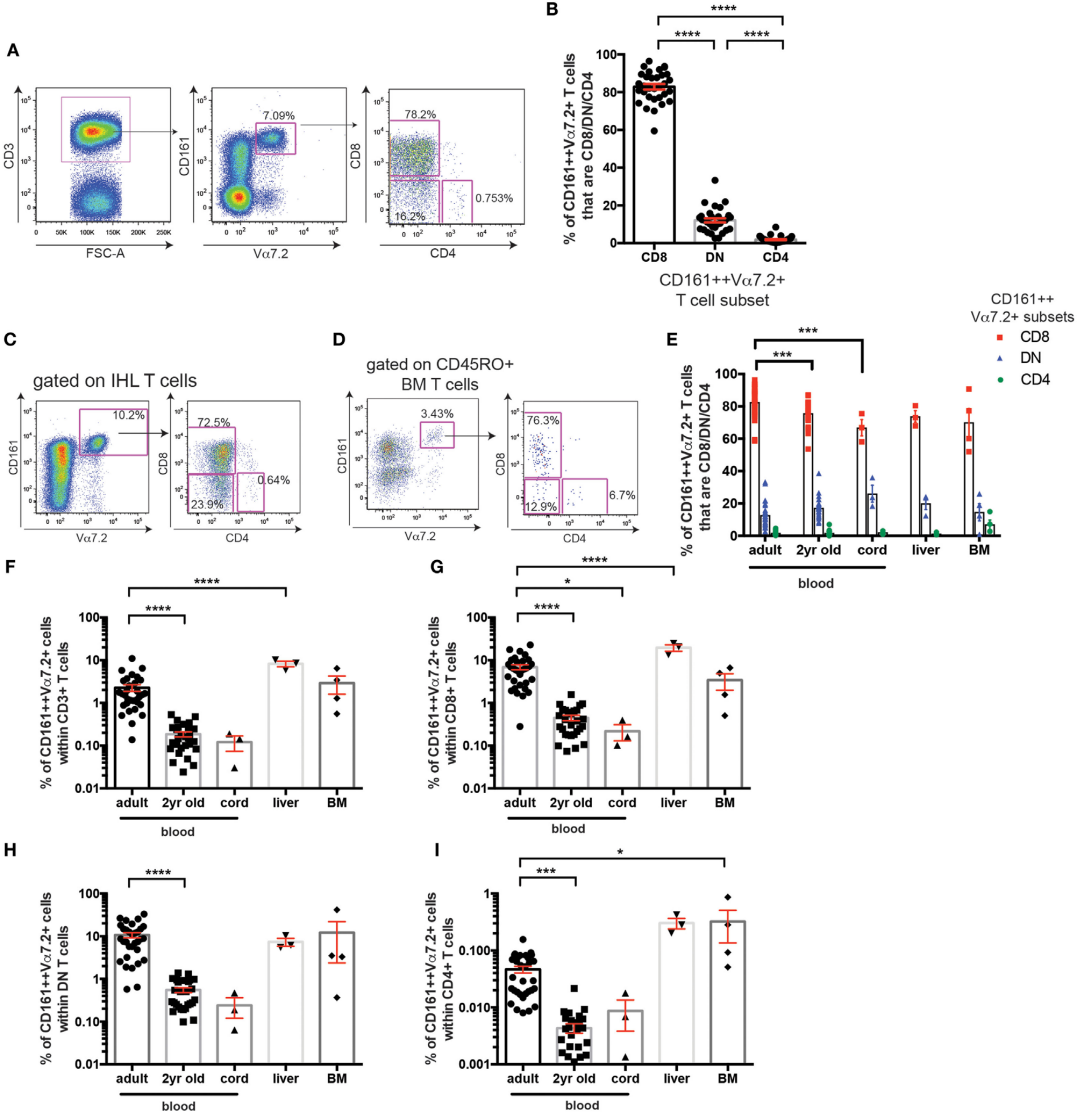
Frequency of CD161++ Vα7.2+ T cell subsets in human blood and tissue. **(A)** Gating strategy used to identify CD161++ Vα7.2+ T cells and their subsets in this study. **(B)** Frequencies of adult peripheral blood CD161++ Vα7.2+ T cells that are CD4+, CD8+ or CD4− CD8− (double negative; DN) (mean ± SEM) (*n* = 32; pooled from four independent experiments). ****P < 0.0001 by one-way ANOVA with Tukey’s multiple comparisons test. **(C,D)** Representative staining showing the expression of coreceptors on CD161++ Vα7.2+ T cells from **(C)** an intrahepatic lymphocyte sample (IHL) and **(D)** bone marrow (BM) sample. **(E)** Frequencies of CD8+ (red), DN (blue) or CD4+ (green) cells within total CD161++ Vα7.2+ T cells in the indicated tissues. *N* = 32 (adult blood), 28 (2-year-old blood), 3 (cord blood), 3 (liver), and 4 (BM). ***P < 0.001 by two-way ANOVA with Dunnett’s multiple comparisons test compared to adult peripheral blood. All other comparisons were non-significant. **(F–I)** The frequency of CD161++ Vα7.2+ T cells within **(F)** total T cells, **(G)** CD8+ T cells, **(H)** DN T cells, or **(I)** CD4+ T cells in the indicated tissues. *N* = 32 (adult blood), 28 (2-year-old blood), 3 (cord blood), 3 (liver), 4 (BM). Bars indicate mean ± SEM. Yr, year. ****P < 0.0001, ***P < 0.001, *P < 0.05 by two-way ANOVA with Dunnett’s multiple comparisons test compared to adult peripheral blood. All other comparisons were non-significant.

The gating strategy used in this study is detailed in Figure 1A. For immunofluorescence staining, dead cells were excluded with the Live/Dead Fixable near-IR dead-cell stain (Invitrogen). For internal staining, cells were fixed with 1% formaldehyde (Sigma Aldrich) and permeabilized with permeabilization buffer (eBioscience). For TF staining, cells were stained with the Foxp3/TF Staining Buffer Set (eBioscience) according to the manufacturers’ protocol. Antibodies used were as follows: anti-CD3 PE-Cy7 or eFluor 450, anti-CD8α eFluor 450 or PerCP-Cy5.5, anti-CD4 PE-Cy7, anti-T-bet PE, anti-Eomes eFluor 660, anti-Eomes FITC, anti-CD69 FITC, anti-IL-17A PE, anti-IL-18Rα PE, anti-CXCR4 PE, and anti-CD94 FITC (eBioscience); anti-CD4 VioGreen, anti-CD8 VioGreen, anti-CD161 PE or APC, anti-IFNγ FITC, anti-CD127 FITC, anti-CD56 APC, anti-CD45 FITC, and anti-TCR γδ T APC-Vio770 (Miltenyi Biotec); anti-promyelocytic leukemia zinc finger (PLZF) APC, anti-macrophage inflammatory protein-1β (MIP-1β) PE, anti-CCR9 PE, and anti-GrA FITC (R&D Systems); anti-CD4 Brilliant Violet 605, anti-CD8 Alexa Fluor 700, anti-CCR7 PE-Cy7, anti-CD56 Brilliant Violet 421, anti-RAR-related orphan receptor γ t (RORγt) PE, anti-CCR6 PerCP-Cy5.5, anti-CCR3 PE, anti-CXCR3 PE, and anti-CD38 FITC (BD Biosciences); anti-CD161 Pacific Blue, anti-Vα7.2 FITC, APC, PE, or PE-Cy7, anti-CD3 PerCP-Cy5.5, anti-TNFα PE-Cy7, anti-Perforin Pacific Blue, anti-IL-4 APC, anti-IL-13 PerCP-Cy5.5, anti-Th-inducing POK (ThPOK) PE, anti-CD45RO PE-Cy7, anti-CD45RA PerCP-Cy5.5, anti-CD62L PE-Cy7, anti-CLA1 Pacific Blue, anti-CCR2 PerCP-Cy5.5, anti-CCR4 PerCP-Cy5.5, anti-CCR5 PerCP-Cy5.5, anti-CCR7 PerCP-Cy5.5, anti-CD25 PE, and anti-CX3CR1 FITC (Biolegend); anti-CD3 Pacific Orange and anti-Granzyme B (GrB) APC (Invitrogen); anti-Granzyme K FITC (Immunotools); and anti-NKG2A (Beckman Coulter).

For MR1-tetramer staining, peripheral blood mononuclear cells (PBMCs) were treated with TruStain FcX Fc Receptor Blocking solution (Biolegend) at 37°C for 20 min, washed with PBS and then stained with antibodies against surface markers and Live/Dead Fixable near-IR dead-cell stain (Invitrogen) in PBS. Cells were then washed and stained with the 5-(2-oxopropylideneamino)-6-d-ribitylaminouracil (5-OP-RU) MR1-tetramer-PE (gifted by Professor James McCluskey or obtained from the NIH Tetramer Core Facility) or Vα7.2-PE antibody for 40 min at room temperature in PBS+ 2% fetal calf serum (FCS). Following further washes, cells were fixed and permeabilized using the Foxp3/TF Staining Buffer Set (eBioscience) following the manufacturer’s instructions (eBioscience), followed by staining with antibodies against intracellular TFs. Where indicated, tetramer+ cells were positively enriched using PE Microbeads (Miltenyi Biotech) before staining.

### *In Vitro* Stimulation of CD161++ Vα7.2+ T Cells

THP1 cells (ECACC, UK) were incubated overnight with paraformaldehyde (PFA)-fixed *Escherichia coli* (*E. coli*; DH5α, Invitrogen) at a ratio of 25 bacteria per cell, or a sterility control. THP1 cells were washed extensively, and PBMCs, intrahepatic lymphocytes, enriched CD8+ T cells, or sorted CD161++ Vα7.2+ T cells were added to the THP1 cells for a 5-h stimulation. Brefeldin A (eBioscience) was added for the final 4 h of the stimulation. Alternatively, for the assessment of degranulation, anti-CD107a-PE-Cy7 (BioLegend) was added from the start of the stimulation. For the assessment of GrB and perforin upregulation, PBMCs were added to *E. coli*-treated THP1 cells for 24 h. MAIT cell apoptosis was detected by Annexin V surface staining (Miltenyi Biotec), in Annexin V binding buffer (Miltenyi Biotec) for 15 min. Staurosporine (Sigma Aldrich) was added as a positive control. Alternatively, MAIT cells were stimulated with IL-12+ IL-18 (both Miltenyi Biotech) at 50 ng/ml for 20 h, and brefeldin A (eBioscience) was added at 3 µg/ml for the final 4 h of the incubation. For blocking experiments, anti-MR1 antibody (gift from Professor Ted Hansen or from Biolegend), anti-IL-12p40/70, anti-IL-18 antibody (both Miltenyi Biotech), or the appropriate isotype controls were added at 10 µg/ml. Anti-CD8α antibody (clone LT8; Novus Biologicals) was added at the indicated concentrations.

### Culture of MAIT Cells in Th2-Skewing Conditions

Peripheral blood mononuclear cells were cultured in RPMI-1640 with 10% human AB serum + penicillin/streptomycin (all from Sigma Aldrich) with 100 ng/ml IL-4 (PreproTech Inc.) and 50 U/ml IL-2 (Miltenyi Biotec) for 6 days, as previously described (31), before washing and stimulation with Phorbol 12-myristate 13-acetate (PMA) and ionomycin (both 250 ng/ml; Sigma Aldrich) for 5 h. Brefeldin A (Sigma Aldrich) was added at 10 µg/ml for the final 4 h of the stimulation.

### IL-12/IL-18 Stimulation of Sorted CD161++ Vα7.2+ T Cell Subsets

Peripheral blood mononuclear cells obtained from leukocyte cones were stained with the following fluorochrome-labeled antibodies; anti-CD8β PE (Beckman Coulter), anti-CD3 V500, anti-CD8α PerCP, anti-CD4 PE-Cy7, anti-CD161 BV421, anti-Vα7.2 FITC and Zombie Green Fixable Viability marker (Biolegend), and Fluorescence-activated cell sorted (FACS) (FACSFusion, BD Biosciences) into indicated MAIT cell subsets. After sorting cells were washed in PBS and incubated for 48 h in RPMI-1640 (Sigma Aldrich) with 10% FCS + penicillin/streptomycin containing IL-12 and IL-18 (both Miltenyi Biotech) at 50 ng/ml along with unsorted PBMCs as negative (unstimulated) and positive (stimulated with IL-12 and IL-18 or PMA/Ionomycin) controls. Supernatants were collected at 48 h and cytokine analysis was performed using the bead-based immunoassay LEGENDplex human Th17 panel (Biolegend) according to the manufacturer’s protocol and analysis performed using LEGENDplex data analysis software (Biolegend).

### Quantitative Real-time PCR

To isolate CD4+ CD161++ Vα7.2+ T cells, CD4+ cells were initially positively selected from freshly isolated PBMCs with the CELLection™ Pan Mouse IgG Kit (Thermo Fisher Scientific), as per the manufacturer’s instructions. Vα7.2+ cells were then positively selected with anti-PE microbeads (Miltenyi Biotec) as per the manufacturer’s instructions. Cells were then stained and CD4+ CD161++ Vα7.2+ CD3+ cells sorted on a FACS Aria (BD Biosciences). CD8+ CD161++ Vα7.2+ CD3+ cells and CD161− Vα7.2− CD3+ cells were sorted directly from PBMC on a FACS Aria (BD Biosciences). DNA was extracted from sorted cells using PureLink Genomic DNA Mini Kit (Life Technologies) as per the manufacturer’s instructions.

The real-time PCR was performed as previously described for on an Viia™ 7 Real-time PCR System (Applied Biosystems) using KAPA PROBE FAST qPCR Master Mix (2X) Universal Kit (Kapa Biosystems), except for the MAIT cell assay (Vα7.2-Jα33/12/20) the following primers and probes were used: Vα7.2 forward primer (TCCTTAGTCGGTCTAAAGGGTACAG), 1 µM; Jα33 reverse primer (CCAGCGCCCCAGATTAA), 200 nM; Jα12 reverse primer (GTCCCACTCCCGAAGATCAATTT) 400 nM; Jα20 reverse primer (TGTGGTTCCGGCTCCAAAG), 400 nM; Vα7.2 probe (6-FAM/AGGTTGCTC/ZEN/CACAGGTAGCTCTAGG/Iowa Black FQ), 400 nM. The efficiency of the Vα7.2-Jα33/12/20 and β-2-microglobulin (β2M) assays were 92.5 and 102.3%, respectively.

### Data Acquisition and Statistical Analysis

Data were collected on the MACSQuant Analyzer (Miltenyi Biotech) and were analyzed using FlowJo v9.8 (TreeStar). All graphs and statistical analyses were completed using GraphPad Prism software Version 6. All data are presented as means with SEM, unless otherwise indicated.

## Results

### Frequencies of CD161++ Vα7.2+ T Cell Subsets in Healthy Blood and Tissue

Mucosal-associated invariant T cells have been previously described to be contained within the CD161++ Vα7.2+ T cell population (4, 11). Gating on all CD3+ CD161++ Vα7.2+ T cells in healthy adult peripheral blood, the majority of CD161++ Vα7.2+ T cells were found to be either CD8+ (82.9 ± 1.5%) or DN (12.1 ± 1.1%), while CD4+ cells only accounted for 1.9 ± 0.3% of total CD161++ Vα7.2+ T cells in adult peripheral blood (Figures 1A,B), in line with previous studies (32, 33).

Next, coreceptor usage of CD161++ Vα7.2+ T cells in BM and liver was compared to that of blood from cord blood, 2-year-old, and adult donors. This revealed that the distribution of CD161++ Vα7.2+ T cell subsets within intrahepatic lymphocytes [intrahepatic lymphocyte sample (IHL); Figure 1C] and in the memory T cell fraction of BM (Figure 1D) is similar to peripheral blood, where the CD8+ CD161++ Vα7.2+ T cell population constitutes the majority of CD161++ Vα7.2+ T cells (Figure 1E). Interestingly, the expression of the CD8 coreceptor by blood CD161++ Vα7.2+ T cells increased with age, when comparing cord, 2-year-old, and adult peripheral blood.

Looking at the frequency of these cells within T cell populations, CD161++ Vα7.2+ T cells accounted for a mean of 2% of adult circulating T cells (Figure 1F), comparable to previous reports (27, 34). Within different T cell subsets in adult peripheral blood, CD161++ Vα7.2+ T cells were found at an average frequency of 7% within CD8+, 11% within DN, and 0.05% of CD4+ T cells (Figures 1G–I). The frequency of CD161++ Vα7.2+ cells within T cells increased with age, as previously described. Interestingly, this increase occurred within CD4+ T cells as well as within CD8+ and DN T cells, suggesting that CD161++Vα7.2+ T cells expand with age regardless of coreceptor expression.

### Cell Surface Phenotype Heterogeneity of Human CD161++ Vα7.2+ T Cells

**Figure 2:**
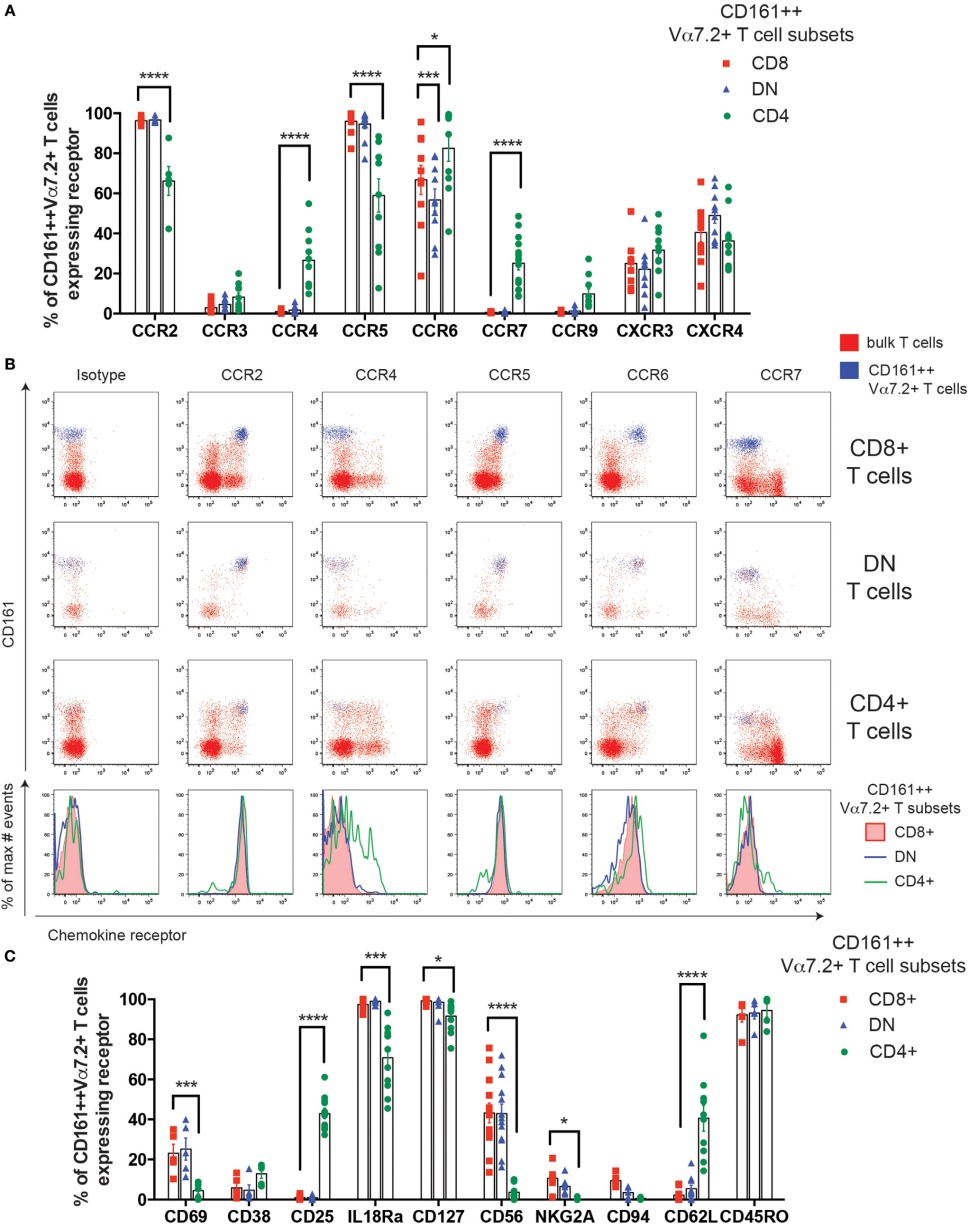
Cell surface phenotype heterogeneity of human CD161++ Vα7.2+ T cells. **(A)** Percentage of CD8+, double negative (DN), and CD4+ CD161++ Vα7.2+ T cells expressing the indicated chemokine receptors. Bars indicate mean ± SEM. ****P < 0.0001, ***P < 0.001, *P < 0.05 by two-way repeated measures ANOVA with Dunnett’s multiple comparisons test, compared to CD8+ CD161++ Vα7.2+ T cells. All other comparisons were not significant (*n* = 5–15; data pooled from independent experiments with biological replicates of 5). **(B)** Representative flow cytometry plots gated on total CD8+ (top row), DN (second row), CD4+ (third row) T cells showing expression of indicated chemokine receptors. Blue = CD161++ Vα7.2+ T cells; red = bulk T cells. Bottom row shows histograms for cells gated on CD8+, DN, or CD4+ CD161++ Vα7.2+ T cells. **(C)** Percentage of CD8+, DN, or CD4+ CD161++ Vα7.2+ T cells expressing the indicated surface receptors. Bars indicate mean ± SEM. ****P < 0.0001, ***P < 0.001, *P < 0.01 by two-way repeated measures ANOVA with Dunnett’s multiple comparisons test, compared to CD8+ CD161++ Vα7.2+ T cells. All other comparisons were not significant (*n* = 5–13).

The previously reported enrichment of CD4+ MAIT cells in lymph nodes in mice (8) suggests that different MAIT cell subsets may traffic to different tissues and, therefore, the expression of chemokine receptors was compared. Blood CD161++ Vα7.2+ T cell subsets were uniformly high for CCR2, CCR5, and CCR6, and had low or little expression of CCR3, CXCR3, and CXCR4 (Figures 2A,B), as previously reported in CD8+ MAIT cells (11). Although some differences in expression were seen for CCR2, CCR5, and CCR6, the largest difference between the subsets was the expression of CCR4 and CCR7. A small proportion (25%) of CD4+ CD161++ Vα7.2+ T cells were found to express CCR4, which was significantly higher than the CD8+ and DN cells. CCR7 expression was similarly only found on a minority (27%) of CD4+ CD161++ Vα7.2+ T cells, while completely absent on CD8+ and DN subsets.

Next, we investigated whether other cell surface markers expressed by CD8+ CD161++ Vα7.2+ T cells were differentially expressed in CD4+ and DN CD161++ Vα7.2+ T cells (Figure 2C). First, a low frequency of CD8+ and DN CD161++ Vα7.2+ T cells expressed the early activation marker CD69, suggesting a level of constitutive activation during circulation. However, the expression of CD69 on CD4+ CD161++ Vα7.2+ T cells was virtually absent. By contrast, CD25 was exclusively found on CD4+ CD161++ Vα7.2+ T cells. Of note, this CD25+ CD4+ CD161++ Vα7.2+ T cell population lacked expression of forkhead box P3 (Foxp3) and was unable to produce transforming growth factor β (data not shown). In addition, the cytokine receptors IL-18R and CD127 were highly expressed in all subsets of CD161++ Vα7.2+ T cells, albeit at a slightly lower frequency in CD4+ CD161++ Vα7.2+ T cells. Lastly, NK cell receptors CD56, NKG2A, and CD94 were consistently expressed on CD8+ and DN CD161++ Vα7.2+ T cells, but CD4+ CD161++ Vα7.2+ T cells lacked their expression, suggesting their lack of cytotoxic potential. Significant phenotypic differences between CD161++ Vα7.2+ T cell subsets are summarized in Table S1 in Supplementary Material.

### CD4+ CD161++ Vα7.2+ T Cells Have Reduced Eomes Expression and Low Cytotoxic Potential

**Figure 3:**
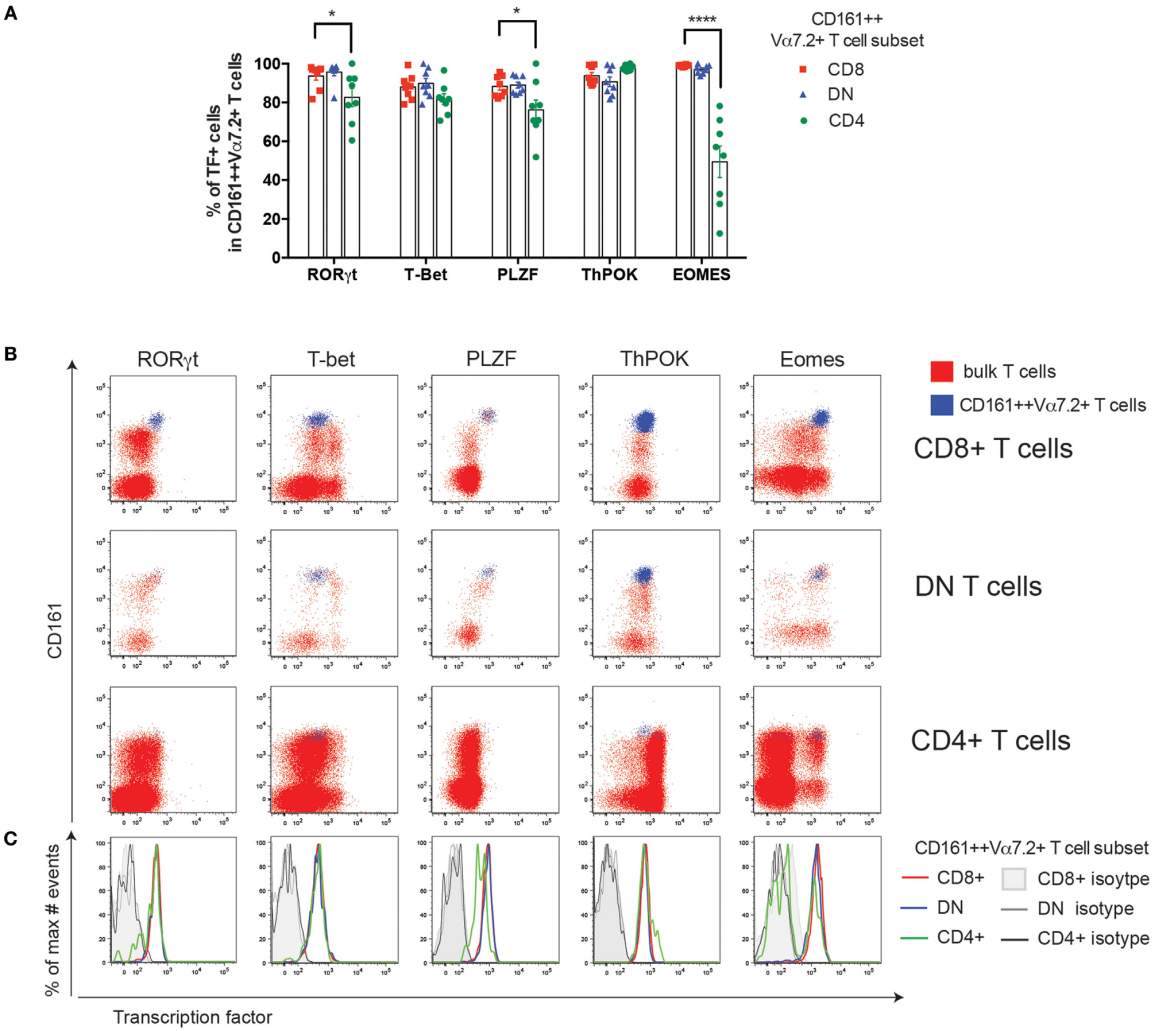

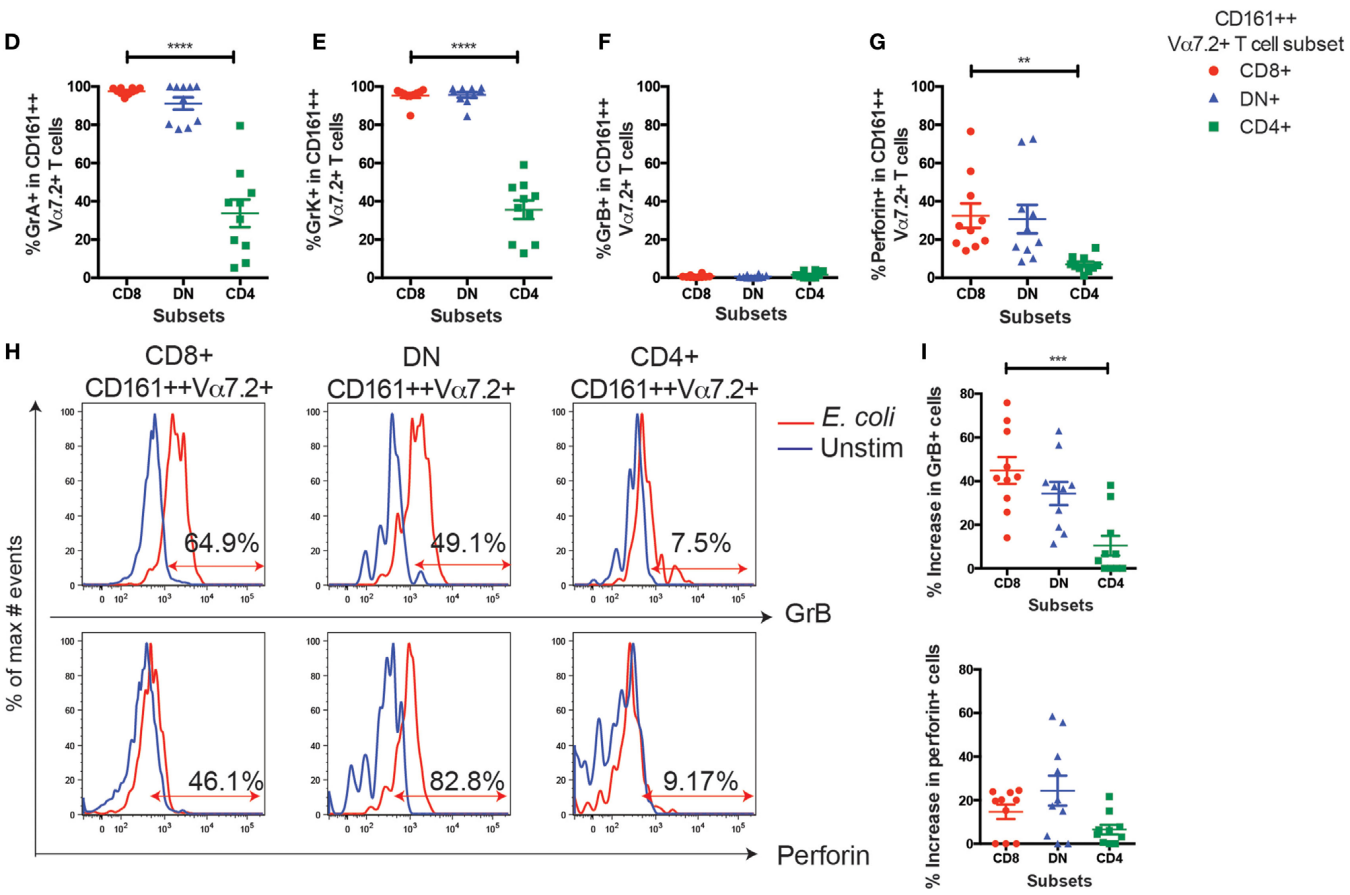
CD4+ CD161++ Vα7.2+ T cells have reduced Eomes expression and low cytotoxic potential. **(A)** The frequency of CD8+, double-negative (DN), and CD4+ CD161++ Vα7.2+ T cells expressing the indicated transcription factors (TFs). Bars indicate mean ± SEM, ****P < 0.0001, ***P < 0.001 by two-way repeated measures ANOVA with Dunnett’s multiple comparisons test, compared to CD8+ CD161++ Vα7.2+ cells. All other comparisons were not significant (*n* = 8; data pooled from two independent experiments). **(B)** Representative flow cytometry plots gated on total CD8+ (top row), DN (second row), CD4+ (third row) T cells showing expression of indicated TFs. Blue = CD161++ Vα7.2+ T cells; red = bulk T cells. **(C)** Overlaid histograms of cells gated on CD8+, CD4+, or DN CD161++ Vα7.2+ T cells showing the expression of the indicated TF, overlaid with their respective isotype control stains in gray as per legend. In all graphs, CD8+ cells are shown in red, DN cells in blue, and CD4+ cells in green. **(D–G)** Frequency of CD8+, DN, CD4+ CD161++ Vα7.2+ T cells expressing: **(D)** granzyme (Gr) A, **(E)** GrK, **(F)** granzyme B (GrB), or **(G)** perforin, showing mean ± SEM. ****P < 0.0001, **P < 0.01 by repeated measures one-way ANOVA with Bonferroni’s multiple comparisons test, compared to CD8+ CD161++ Vα7.2+ T cells (*n* = 10; data pooled from two independent experiments). **(H,I)** Upregulation of GrB and perforin in CD161++ Vα7.2+ T cell subsets following stimulation with *Escherichia coli*-treated THP1 cells for 24 h. **(H)** Representative staining showing GrB and perforin upregulation in CD161++ Vα7.2+ T cells. Percentages indicate frequency of cells expressing indicated effector molecule, based on isotype control. **(I)** The fraction of cells expressing GrB or perforin at resting conditions was subtracted to show increases in GrB or perforin expression after *E. coli* stimulation. ***P < 0.001 by repeated measures one-way ANOVA with Bonferroni’s multiple comparisons test, compared to CD8+ CD161++ Vα7.2+ T cells (*n* = 10; data pooled from two independent experiments). All other comparisons were non-significant.

Next we looked at the differences in TF expression in CD161++ Vα7.2+ T cell subsets. All three subsets of blood CD161++ Vα7.2+ T cells expressed high levels of RORγt and were found to be T-betlow (T-box expressed in T cells; Figures 3A–C), as previously described (35), although CD4+ CD161++ Vα7.2+ T cells had a slightly lower frequency of cells expressing RORγt compared to CD4− CD161++ Vα7.2+ T cells. In addition, CD8+ MAIT cells have also been shown to express the master regulator of innate-like T cells, PLZF, at high levels (11, 28). We found that all three subsets uniformly expressed PLZF, but the frequency of cells expressing PLZF was slightly, but significantly lower in the CD4+ CD161++ Vα7.2+ T cells compared to the CD8+ CD161++ Vα7.2+ T cells (Figures 3A,B).

Next, the expression of ThPOK was examined, a TF associated with CD4 lineage commitment during thymic development (36), which also suppresses RORγt expression in iNKT cells (37). We found that all three subsets of CD161++ Vα7.2+ T cells expressed ThPOK (Figures 3A,B). Interestingly, when comparing the geometric mean fluorescence intensity of ThPOK expressed by CD161++ Vα7.2+ T cells to conventional CD4+, CD8+, or DN T cells (Vα7.2−), CD161++ Vα7.2+ T cells were found to consistently express higher levels of ThPOK compared to conventional CD8+ and DN T cells, while expressing lower levels compared to conventional CD4+ T cells (Figures S1A,B in Supplementary Material), showing that CD161++ Vα7.2+ T cells are ThPOKlow.

Lastly, we found that CD4+ CD161++ Vα7.2+ T cells expressed significantly less Eomesodermin (Eomes) compared to CD8+ and DN CD161++ Vα7.2+ T cells in frequency (Figure 3A), with an average of 50% of CD4+ CD161++ Vα7.2+ T cells expressing Eomes compared to CD8+ and DN CD161++ Vα7.2+ T cells, in which the majority of cells expressed Eomes. Previously, we have shown that CD8+ CD161++ Vα7.2+ T cells express granzyme A (GrA), GrK, and low levels of perforin at resting conditions (38). The greatly lower expression of Eomes by CD4+ CD161++ Vα7.2+ T cells suggests that these cells may have low cytotoxic potential. Indeed, we found that DN CD161++ Vα7.2+ T cells expressed GrA, GrK, and perforin at comparable frequencies to CD8+ cells, but significantly reduced frequencies of CD4+ CD161++ Vα7.2+ T cells expressed GrA, GrK, and perforin compared to CD8+ cells (Figures 3D–G). All subsets expressed little GrB at resting conditions.

CD8+ MAIT cells can rapidly upregulate GrB and perforin following bacterial stimulation, which arms these cells with the ability to efficiently kill target cells (35, 38). To investigate whether CD4+ CD161++ Vα7.2+ T cells can become killers, CD161++ Vα7.2+ T cells were stimulated with *E. coli*-treated THP1 cells for 24 h and GrB and perforin expression was analyzed. As shown in Figures 3H,I, the increase in the fraction of CD4+ CD161++ Vα7.2+ T cells expressing GrB was significantly lower compared to CD8+ CD161++ Vα7.2+ T cells. There was no significant difference between all subsets in their ability to upregulate perforin in response to bacterial stimulation.

### CD8+ and DN CD161++ Vα7.2+ T Cells Have a Higher Capacity to Secrete Th1 Cytokines

**Figure 4:**
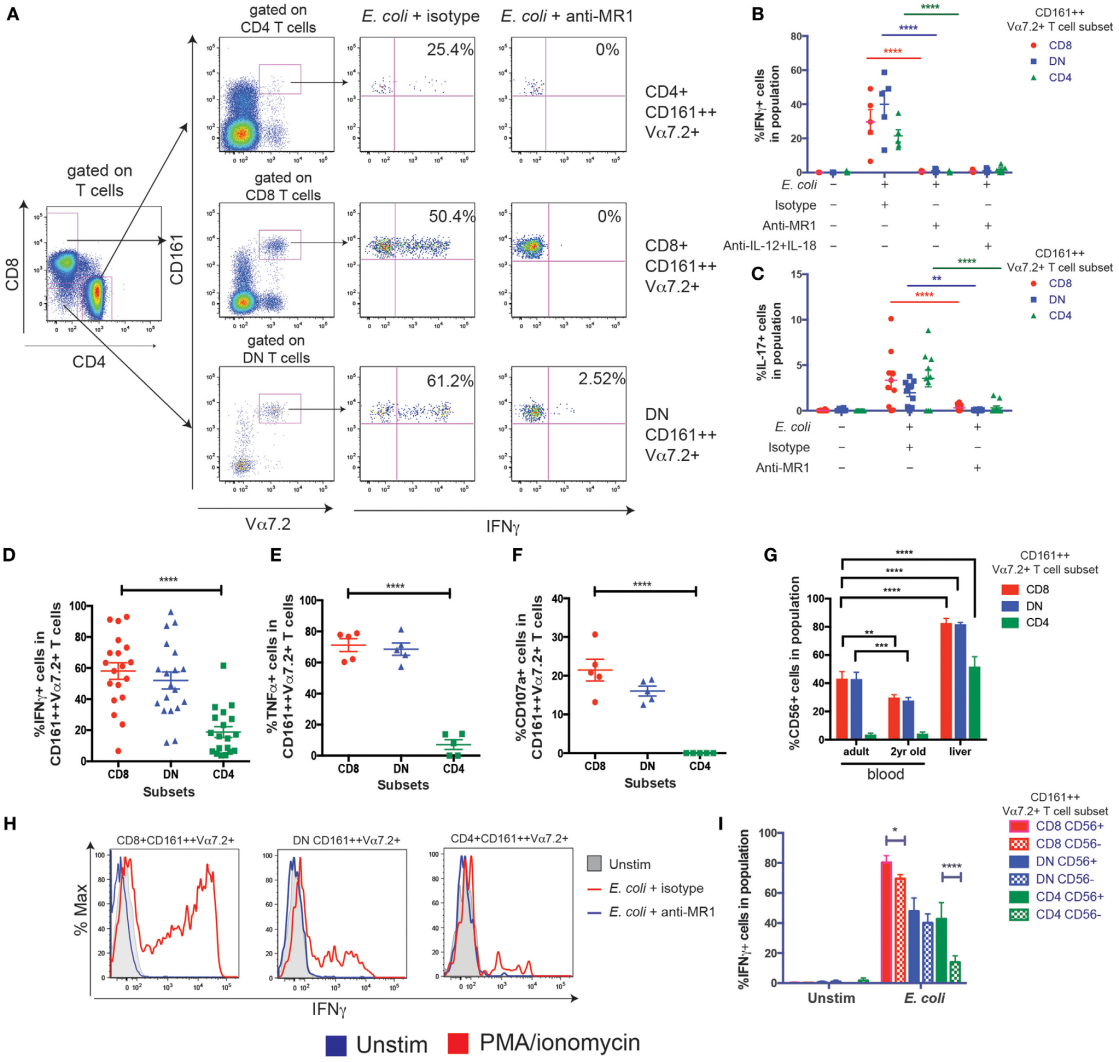

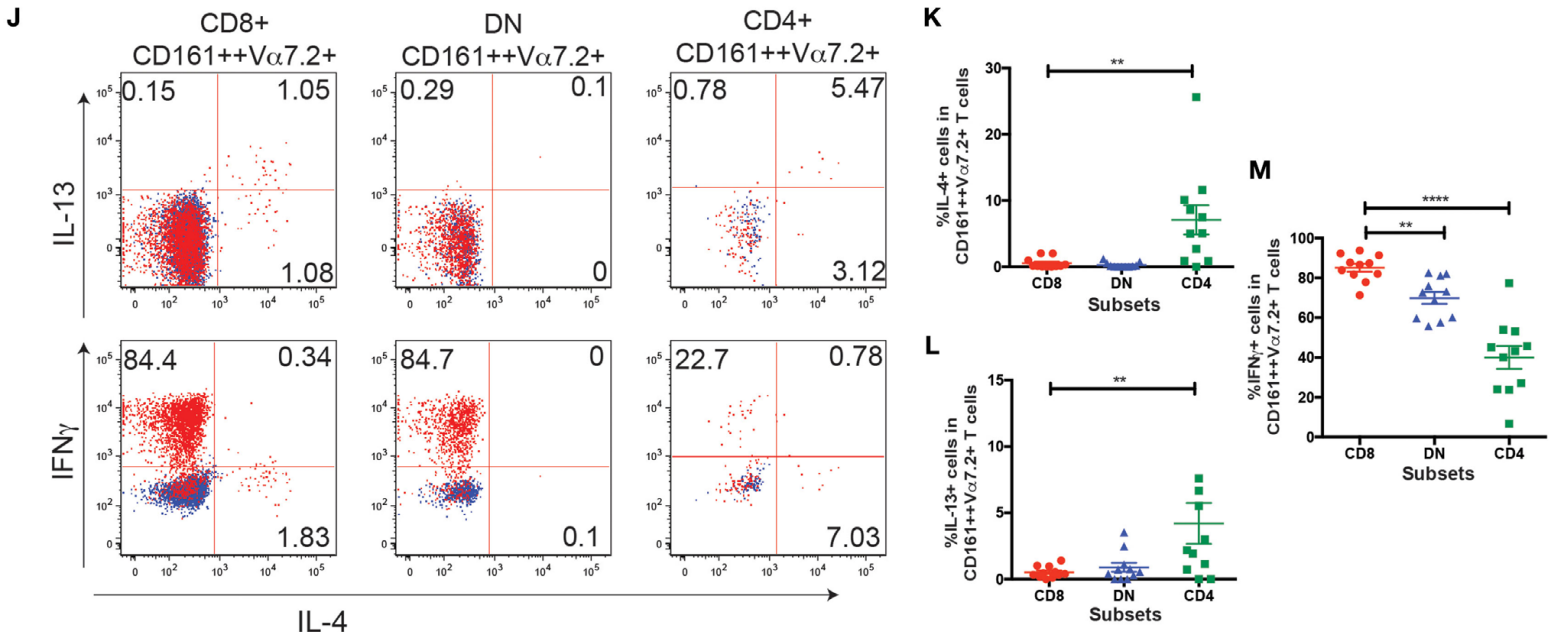
CD8+ and double-negative (DN) CD161++ Vα7.2+ T cells have a higher capacity to secrete T helper 1 cytokines. **(A–F)** THP1 cells were cultured with *Escherichia coli* overnight before co-culturing with peripheral blood mononuclear cells (PBMCs) for 5 h. **(A)** PBMCs were cultured for 5 h with *E. coli*-treated THP1 cells in the presence or absence of anti-MHC class I-related protein 1 (MR1) blocking antibody. Representative staining is shown. **(B)** Frequency of CD161++ Vα7.2+ T cells expressing IFNγ **(B)** or interleukin-17 (IL-17) **(C)** in response to *E. coli*-treated THP1s in the presence or absence of indicated blocking antibodies against MR1, IL-12, IL-18, or isotype controls for 5 h. Bars indicate mean ± SEM (*n* = 5 for IFNγ, *n* = 10 for IL-17). ****P < 0.0001, **P < 0.01 by two-way ANOVA with Dunnett’s multiple comparisons test, compared to *E. coli* + isotype for each subset. Comparison with no *E. coli* not shown. **(D–F)** Frequency of CD8+, DN, or CD4+ CD161++ Vα7.2+ T cells expressing **(D)** IFNγ **(E)** TNFα **(F)** CD107a in response to *E. coli*-treated THP1s. ****P < 0.0001 by repeated measures one-way ANOVA with Dunnett’s multiple comparisons test, compared to CD8+ CD161++ Vα7.2+ T cells. All other comparisons were non-significant (*n* = 19 for IFNγ, from three independent experiments; *n* = 5 for all others). **(G)** Frequencies of CD56-expressing CD161++ Vα7.2+ T cells within blood and liver, according to coreceptor expression. ****P < 0.0001, ***P < 0.001, **P < 0.01 by two-way ANOVA with Dunnett’s multiple comparisons test, compared to adult blood within each subset. Other comparisons were non-significant. **(H,I)** Intrahepatic lymphocyte cells were cultured with *E. coli*-treated THP1 cells for 5 h. Representative staining **(H)** of IFNγ-expressing intrahepatic CD161++ Vα7.2+ T cells, in the presence or absence of an anti-MR1 blocking antibody added at 10 μg/ml: (blue line) or isotype control (red line). **(I)** Expression of IFNγ from indicated intrahepatic CD161++ Vα7.2+ T cell subsets according to CD56 expression. ****P < 0.0001, *P < 0.05 by two-way repeated measures ANOVA with Tukey’s multiple comparisons test, comparing CD56+ and CD56− cells within each CD161++ Vα7.2+ T cell subset (*n* = 3). **(J–M)** PBMCs were cultured in Th2-skewing conditions [interleukin-4 (IL-4) + IL-2, see Materials and Methods] and restimulated with phorbol 12-myristate 13-acetate (PMA)/ionomycin for 5 h. **(J)** Representative plots showing IL-4, IL-13, and IFNγ production in response to PMA/ionomycin from CD161++ Vα7.2+ T cells. **(K–M)** Percentage of CD161++ Vα7.2+ T cells expressing **(K)** IL-4 **(L)** IL-13 **(M)** IFNγ. ****P < 0.0001, **P < 0.01 by repeated measures one-way ANOVA with Dunnett’s multiple comparisons test, compared to CD8+ CD161++ Vα7.2+ T cells (*n* = 11; data pooled from two independent experiments). All other comparisons were not significant.

Next, to determine the functional differences between the three CD161++ Vα7.2+ T cell subsets, *E. coli*-treated THP1 cells were used to probe the MR1-dependent activation of CD161++ Vα7.2+ T cells. THP1 cells were cultured with PFA-fixed *E. coli* overnight before washing and co-culturing with PBMCs for 5 h. We did not observe a significant difference in the expression of the CD8 or CD4 coreceptors or proportions of CD8, DN, and CD4+ CD161++ Vα7.2+ T cells following *E. coli* stimulation due to change in coreceptor expression (Figures S2A–C in Supplementary Material) in control experiments. There was a clear production of interferon-γ (IFNγ) from all three subsets of CD161++ Vα7.2+ T cells after stimulation with *E. coli*-treated THP1 cells, which was completely blocked by the addition of an anti-MR1 blocking antibody (Figures 4A,B), consistent with previous work in this time frame (39). IL-17 was expressed by all CD161++ Vα7.2+ T cell subsets and its expression was also completely MR1 dependent (Figure 4C).

Using this MR1-dependent model of MAIT cell activation, the frequency of cytokine-producing cells within each subset was further compared. There was no difference in the frequency of CD8+ and DN CD161++ Vα7.2+ T cells producing IFNγ, but the frequency of CD4+ CD161++ Vα7.2+ T cells producing IFNγ and tumor necrosis factor-α (TNFα) was significantly lower (Figures 4D,E). Importantly, while similar frequencies of CD8+ and DN CD161++ Vα7.2+ T cells were able to degranulate (Figure 4F), there was a complete lack of degranulation from CD4+ CD161++ Vα7.2+ T cells.

In addition, as MAIT cells can also be activated in an MR1-independent manner through cytokines (39), PBMCs were stimulated overnight with IL-12 + IL-18, and the expression of IFNγ by MAIT cell subsets was compared. Only a mean of 5% of CD4+ CD161++ Vα7.2+ T cells produced IFNγ (Figures S2D in Supplementary Material), compared to the high proportion of IFNγ+ CD8+ and DN CD161++ Vα7.2+ T cells following IL-12 + IL-18 stimulation. This was also confirmed using a LEGENDplex bead-based immunoassay of sorted CD161++ Vα7.2+ T cell subsets (Figures S2E,F in Supplementary Material).

Together, these results suggest that blood CD4+ CD161++ Vα7.2+ T cells have a low capacity to secrete Th1 cytokines compared to other subsets of CD161++ Vα7.2+ T cells, and do not degranulate after short-term activation. Interestingly, within the liver, a greater proportion of all CD161++ Vα7.2+ T cell subsets expressed CD56 (Figure 4G). Intrahepatic CD8+ CD161++ Vα7.2+ T cells had the highest MR1-dependent IFNγ expression in response to *E. coli*-pulsed THP1 cells compared to DN and CD4+ CD161++ Vα7.2+ T cells (Figure 4H), and CD56 expression was associated with a higher frequency of IFNγ+ CD161++ Vα7.2+ T cells (Figure 4I). This was especially true within the intrahepatic CD4+ subset, suggesting that CD56 expression is associated with a greater Th1 response from CD4+ CD161++ Vα7.2+ T cells.

Next, the potential of CD161++ Vα7.2+ T cells to produce Th2 cytokines was investigated. PBMCs were cultured in Th2-skewing conditions (IL-4 + IL-2) and restimulated with PMA/ionomycin for 5 h (Figure 4J). Blood CD4+ CD161++ Vα7.2+ T cells contained a significantly higher fraction of cells expressing IL-4 and IL-13 compared to the CD8+ and DN CD161++ Vα7.2+ T cells (Figures 4K,L). In turn, CD4+ CD161++ Vα7.2+ T cells produced significantly less IFNγ compared to their CD8+ and DN counterparts in response to PMA/ionomycin (Figure 4M), in line with their reduced capacity to produce IFNγ in previous assays. Interestingly, low production of IL-4 and IL-13 was unique to CD161++ Vα7.2+ T cells within the CD8+ T cell population, as other populations within CD8+ T cells were able to produce IL-4 and IL-13 to similar or greater levels compared to CD4+ T cells (Figures S2G–I in Supplementary Material). A similar trend was visible within the DN T cell population, whereas within the CD4+ T cell population, the ability of CD161++ Vα7.2+ T cells to produce IL-4 and IL-13 was comparable to conventional CD4+ T cell populations. This suggests that Th2 cytokine production may be uniquely suppressed within CD8+, and to a lesser extent, DN CD161++ Vα7.2+ T cells, compared to CD4+ CD161++ Vα7.2+ T cells.

### MR1-Tetramer-Sorted CD161++ CD4+ MAIT Cells Have Lower Eomes and PLZF Expression

**Figure 5:**
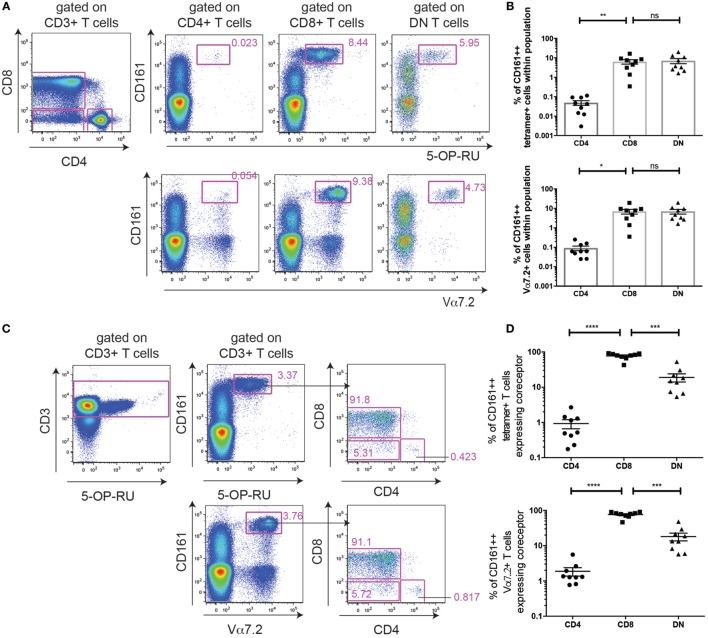
MHC class I-related protein 1 (MR1)-tetramer staining identifies mucosal-associated invariant T cells within the CD161++ Vα7.2+ T cell population. **(A)** Representative plots showing cells staining for 5-OP-RU MR1-tetramer (top) or Vα7.2 antibody (bottom) within CD4+, CD8+, and double-negative (DN) T cells. Cells are from the same donor, stained in parallel. Frequency of cells within CD4+, CD8+, or DN T cells shown in **(B)**. **P < 0.01 or *P < 0.05, or non-significant = ns by repeated measures one-way ANOVA with Dunnett’s multiple comparisons test, compared to CD8+ T cells (*n* = 9). **(C)** Same cells as **(A)**, showing coreceptor expression on CD161++ T cells stained with 5-OP-RU MR1-tetramer (top) or Vα7.2 antibody (bottom). **(D)** Coreceptor expression on CD161++ MR1-tetramer+ cells (top) and CD161++ Vα7.2+ T cells (bottom). ****P < 0.0001, ***P < 0.001 by repeated measures one-way ANOVA with Dunnett’s multiple comparisons test, compared to CD8+ T cells (*n* = 9).

Next, to confirm the MR1-reactivity of the cells, we used 5-OP-RU-MR1-tetramers to define MAIT cells (Figures 5A–D). Although we had found that the Vα7.2-Jα33/12/20 gDNA rearrangement was 339-fold enriched in the CD4+ CD161++ Vα7.2+ T cells compared to Vα7.2−CD161−T cells (40), and was not significantly different to the abundance of the Vα7.2-Jα33/12/20 rearrangement in CD8+ CD161++ Vα7.2+ T cells (Figure S3 in Supplementary Material), the MR1-tetramer has recently become widely available and is the most accurate method of defining MAIT cells. We found that the frequency of CD161++ MR1-tetramer+ cells that were CD8+ (76.9 ± 5.1%) was comparable with the frequency of CD8+ cells within CD161++ Vα7.2+ T cells (76.7 ± 5%). DN CD161+ MR1-tetramer+ T cells were similarly comparable with DN CD161++ Vα7.2+ T cells (19.0 vs. 18.2%; Figures 5C,D). The frequency of CD161++ Vα7.2+ T cells that were CD4+ (1.9 ± 0.5%), however, was slightly higher than the frequency of CD161++ MR1-tetramer+ T cells that were CD4+ (0.9 ± 0.3%) in the same donors. This suggests that a proportion of the CD4+ CD161++ Vα7.2+ T cells may be CD161++ CD4+ T cells that express the TCRα chain Vα7.2 but have a different specificity.

**Figure 6:**
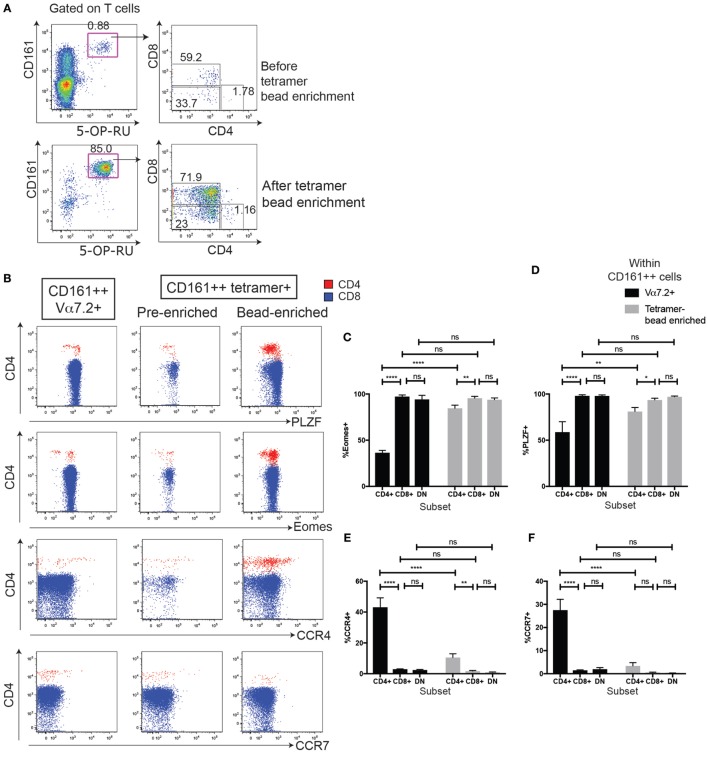

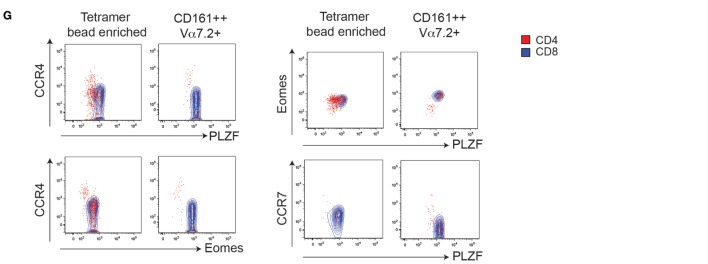
MHC class I-related protein 1 (MR1)-tetramer bead enrichment shows that CD161++ CD4+ mucosal-associated invariant T (MAIT) cells have lower Eomes and promyelocytic leukemia zinc finger (PLZF) expression. **(A)** Representative plots showing the enrichment of MAIT cells by magnetic bead enrichment using the MR1-tetramer. **(B)** Expression of PLZF, Eomes, and CCR4 on CD161++ MR1-tetramer+ cells according to coreceptor expression, before or after tetramer enrichment (*n* = 9). **P < 0.01, *P < 0.05 by repeated measures one-way ANOVA with Dunnett’s multiple comparisons test. Fluorescence-activated cell sorted plots show the expression of the indicated marker on cells gated on either CD161++ MR1-tetramer+ cells before or after tetramer enrichment from the same donor and gated on CD4+ (red) or CD8+ (blue) cells. Plots gated on CD161++ Vα7.2+ T cells shown for comparison. **(C–F)** Comparison of the frequency of cells expressing Eomes **(C)**, PLZF **(D)**, CCR4 **(E)**, and CCR7 **(F)** within CD161++ Vα7.2+ T cells (black) and CD161++ MR1-tetramer+ cells within MR1-tetramer enriched cells (gray). ****P < 0.0001, **P < 0.01, *P < 0.05, and ns = non-significant by two-way ANOVA with Dunnett’s multiple comparisons test comparing each subset to CD8 cells, or by two-way ANOVA with Sidak’s multiple comparisons test comparing Vα7.2+ cells with MR1-tetramer+ cells. **(G)** Coexpression of PLZF, Eomes, and CCR4 on cells gated on either CD161++ MR1-tetramer+ cells or CD161++ Vα7.2+ T cells, and then gated on CD4+ (red) or CD8+ (blue) cells.

In order to improve the staining and phenotype, the CD4+ MR1-tetramer+ cells, we sorted for MAIT cells using MR1-tetramer bead enrichment and investigated whether characteristics of CD4+ CD161++ Vα7.2+ T cells were shared with CD4+ MR1-tetramer+ cells (Figure 6A). We focused on four phenotypic markers that were differentially expressed in CD4+ CD161++ Vα7.2+ T cells compared to CD8+ and DN cells (summarized in Table 1). This analysis showed that in each case, there was a significant difference between the frequency of marker expression in CD4+ CD161++ Vα7.2+ T cells compared to sorted CD4+ MR1-tetramer+ T cells due to some of the CD4+ CD161++ Vα7.2+ T cell population being non-MAIT, MR1-tetramer-negative cells (Figures 6C–F). However, we found that the frequency of Eomes+ cells as well as PLZF+ cells was significantly lower in CD4+ MR1-tetramer+ cells compared to CD8+ and DN MR1-tetramer+ cells, as found in CD4+ CD161++ Vα7.2+ T cells (Figures 6B–D). A small proportion of CD4+ MR1-tetramer+ cells also expressed CCR4, similar to CD4+ CD161++ Vα7.2+ T cells, although at a lower frequency compared to the frequency of CCR4+ cells within CD4+ CD161++ Vα7.2+ T cells and the expression was heterogeneous (Figure 6E). CCR7 expression, by contrast, was significantly different between CD161++ Vα7.2+ T cell subsets but not between MR1-tetramer sorted cells (Figure 6F). Of note, in both CD161++ MR1-tetramer+ T cells and CD161++ Vα7.2+ T cells, Eomes and PLZF were coexpressed, while Eomes and PLZF were expressed in a mutually exclusive manner with CCR4 (Figure 6G). This suggests that although some CD4+ CD161++ Vα7.2+ T cells did not stain for the MR1-tetramer, the pattern of expression of Eomes, PLZF, and CCR4 is shared between CD4+ CD161++ Vα7.2+ T cells and CD4+ MR1-tetramer+ cells. Further analysis of the coexpression of Eomes and PLZF with the other markers differentially expressed between CD4+ and CD4−CD161++ Vα7.2+ T cells showed that CD56, Granzyme A and IFNγ expression is restricted to Eomes+ cells, particularly within CD4+ CD161++ Vα7.2+ T cells (Figures S4A,B in Supplementary Material).

**Table 1:** Summary of markers that are significantly differentially expressed between CD4, CD8, and double-negative (DN) subsets of CD161++ Vα7.2+ T cells, or MR1-tetramer+ MAIT cells.

	CD161++Vα7.2+ T cells			MR1-tetramer+ MAIT cells		
Marker	CD4	CD8	DN	CD4	CD8	DN
CCR4	+	−	−	+	−	−
CCR7	+	−	−	−	−	−
PLZF	++	+++	+++	++	+++	+++
Eomes	+	+++	+++	+	+++	+++

*PLZF, promyelocytic leukemia zinc finger; Eomes, eomesdermin; +++, high expression; ++, medium expression; +, low expression; −, no expression*.

### CD8 Coreceptor Blockade Reduces CD8+ MAIT Cell Activation by *E. coli*-Treated THP1 Cells

**Figure 7:**
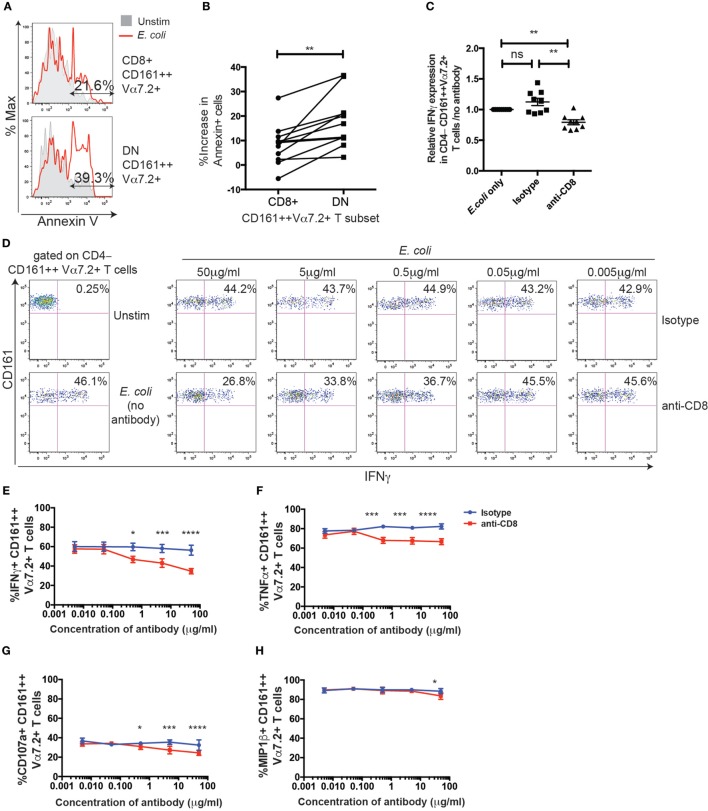
CD8 coreceptor blockade reduces CD8+ CD161++ Vα7.2+ T cell activation by *Escherichia coli*-treated THP1 cells. **(A)** Representative flow cytometry plot of Annexin V staining of CD8+ (top) or double-negative (DN) (bottom) CD161++ Vα7.2+ T cells following incubation with *E. coli*-treated THP1 cells for 5 h. Total % of Annexin V+ cells after *E. coli* stimulation in indicated populations are shown. **(B)** Percentage increase in the frequency of Annexin V+ CD161++ Vα7.2+ T cells compared to unstimulated cells. **P < 0.01 by paired *t*-test (*n* = 11; data from two independent experiments). **(C)** Data from two independent experiments showing the frequency of CD4−CD161++ Vα7.2+ T cells expressing IFNγ in response to *E. coli*-treated THP1s in the presence or absence of blocking antibodies against CD8 (5 µg/ml), or isotype control for 5 h (*n* = 9). Data are presented as relative IFNγ expression compared with that of co-cultures in the absence of any antibodies. **P < 0.01 by one-way ANOVA with Dunnett’s multiple comparisons test, compared to CD161++ Vα7.2+ T cells cultured in the absence of any antibodies. **(D)** Representative plots of IFNγ expression in CD4− CD161++ Vα7.2+ T cells cultured in the presence of increasing concentrations of blocking antibody against CD8, or isotype control, or in the absence of antibodies (far left column). **(E–H)** Frequency of CD4− CD161++ Vα7.2+ T cells expressing **(E)** IFNγ, **(F)** TNFα, **(G)** CD107a, or **(H)** macrophage inflammatory protein-1β (MIP-1β) in the presence (red) or absence (blue) of a blocking antibody against CD8. Each symbol and bar represents the mean ± SEM (*n* = 5). ****P < 0.0001, ***P < 0.001, *P < 0.05 by two-way repeated measures ANOVA with Dunnett’s multiple comparisons test, compared to CD161++ Vα7.2+ T cells cultured at the corresponding concentration of isotype control antibody. All other comparisons were non-significant.

Thus far, no major differences could be observed between the CD8+ and DN MAIT/CD161++Vα7.2+ T cells in phenotype and function in our model. Given that the ratio of DN MAIT cells to CD8+ MAIT cells has been reported to increase with age (15, 16), we asked whether there may be difference in survival after antigen-dependent activation. Therefore, PBMCs were added to *E. coli*-treated THP1 cells and expression of the apoptosis marker Annexin V on CD161++ Vα7.2+ T cells was assessed (Figure 7A). This in fact showed that the increase in Annexin V-expressing cells following stimulation was significantly greater in DN CD161++ Vα7.2+ T cells, compared to CD8+ cells (Figure 7B). Thus, although both CD8+ and DN CD161++ Vα7.2+ T cells are similarly activated in an MR1-dependent manner in our model, the CD8+ CD161++ Vα7.2+ T cell population may be protected from activation-induced cell death.

To further investigate the role of the CD8 coreceptor in the function of MAIT/CD161++ Vα7.2+ T cells, PBMCs were incubated with *E. coli*-treated THP1 cells over 5 h in the presence or absence of an anti-CD8α blocking antibody (clone LT8). Due to the residual loss of CD8 staining from the presence of the anti-CD8 antibody, CD4−CD161++ Vα7.2+ T cells were gated in the following experiments. We found a dose-dependent reduction in the frequency of IFNγ+ CD161++ Vα7.2+ T cells with CD8 coreceptor blockade, compared to the isotype control (Figures 7C,D). This effect was also seen when measuring different outputs of TCR signaling, namely TNFα, CD107a, and macrophage inflammatory protein-1β (Figures 7E–H). Interestingly, the degree to which CD8-coreceptor blockade affected the response of CD161++ Vα7.2+ T cells differed depending on the response measured, with the inhibitory effect of CD8 coreceptor blockade increasing as follows: MIP-1β < CD107a < TNFα ≤ IFNγ. When magnetically enriched CD8+ T cells and the CD8-depleted population were cultured with *E. coli*-treated THP1s in the presence of the anti-CD8 blocking antibody, there was a significant reduction in the frequency of IFNγ+ and TNFα+ cells in the CD8+ CD161++ Vα7.2+ T cells but not in the DN CD161++ Vα7.2+ T cell population (Figure S5 in Supplementary Material), confirming the specificity of the anti-CD8 blocking antibody.

## Discussion

In this study, we have performed a detailed comparison of the CD4+, CD8+, and DN CD161++ Vα7.2+ T cell subsets in parallel, in terms of their phenotype, cytokine secretion, cytotoxicity, and TF expression, which has highlighted their distinct and overlapping characteristics.

First, we found that CD4+ CD161++ Vα7.2+ T cells have a lower frequency of cells expressing Eomes and PLZF compared to their CD4− counterparts—a feature shared with CD4+ MR1-tetramer+ cells. Interestingly, there was a complete lack of degranulation by CD4+ CD161++ Vα7.2+ T cells in response to *E. coli* stimulation, while Eomes+ CD4+ CD161++ Vα7.2+ T cells were enriched for CD56+ and GrA+ cells (Figures S4B,C in Supplementary Material). Thus, CD4+ CD161++ Vα7.2+ T cells may have lower cytotoxic capacity compared to CD4− subsets due to their reduced expression of Eomes. In addition to their lower cytotoxic potential, CD4+ CD161++ Vα7.2+ T cells had a lower capacity to produce Th1 cytokines, and IFNγ expression from CD4+ CD161++ Vα7.2+ T cells was restricted to Eomes+ cells. The CD4+ subset of cells also had a higher capacity to secrete IL-4 and IL-13 compared to their CD4− counterparts, which is in line with the fact that overexpression of Runx3, the silencer of CD4 expression during T cell development, induces Eomes and suppresses IL-4 secretion (41). Although the proportion of CD161++ Vα7.2+ T cells secreting Th2 cytokines was generally low compared to Th1 cytokine-producing CD161++ Vα7.2+ T cells, this supports recent findings in Vα19-Jα33 TCR-transgenic mice showing that CD4+ MAIT cells were the dominant producers of IL-4 in response to TCR stimulation (42).

Interestingly, all subsets of intrahepatic CD161++ Vα7.2+ T cells expressed CD56 at high levels, which was associated with a higher effector function, especially in the CD4+ subset, secreting abundant IFNγ in response to MR1-presented antigen. As CD56 expression has been previously associated with increased cytotoxic effector function of T cells (43, 44), CD4+ CD161++ Vα7.2+ T cells may also have heterogeneous cytotoxic capacities depending on the tissue they reside in. Increased CD56 expression in T cells and NK cells have been reported in *in vitro* cultures of cells with common γ-chain cytokines (43, 45). It is, therefore, possible that the intrahepatic cytokine milieu upregulates CD56 expression on all MAIT cell subsets and lowers their activation threshold and/or skews them toward a Th1 response. Indeed, intrahepatic lymphocytes are dominated by rapidly acting innate cells, including MAIT cells, γδ T cells, NK cells, and T cells expressing NK receptors, e.g., CD56, and constitutive expression of cytokines, such as IL-15 (46) and IL-7 (30), may activate and induce CD56 upregulation in MAIT cells.

In addition, we found that all three CD161++ Vα7.2+ T cell subsets expressed ThPOK, the master regulator of the CD4 lineage (47), at an intermediate level (ThPOKlow). Whether this TF may be expressed at a higher level in the CD4+ subset of CD161++ Vα7.2+ T cells during their development is unknown. Interestingly, a recent report showed that developing MAIT cells in the thymus transition from mostly CD4+ or CD4+ CD8+ to DN or CD8+ cells, which does not occur in PLZF-deficient mice (48). This suggests that the expression of coreceptors during development may be determined by the maturation status of MAIT cells, rather than CD4/CD8 lineage commitment signals regulated by TFs such as ThPOK. Of note, ThPOK also negatively regulates Th17 differentiation and, thus, only cells that express low levels of ThPOK are permissive for the differentiation of type-17 iNKT cells (20, 37, 49). As all subsets of CD161++ Vα7.2+ T cells expressed IL-17 to a similar level, CD161++ Vα7.2+ T cells may also express a reduced amount of ThPOK that is permissive for the expression of RORγt.

The most accurate method of defining a MAIT cell currently available is by identification of cells that bind the MR1-tetramer. The MR1-tetramer has been developed (4, 5) but was not widely available until recently, and so the most commonly used method for identifying MAIT cells has previously been to look at CD161++ Vα7.2+ T cells. These cells have been shown to overlap with CD161++ MR1-tetramer stained cells (4), particularly in CD8+ and DN cells (27). In this study, we show that although the MAIT cell TCR Vα7.2-Jα33/12/20 is enriched within the CD4+ CD161++ Vα7.2+ T cell population, not all CD4+ CD161++ Vα7.2+ T cells could be detected by the 5-OP-RU-loaded MR1-tetramer. This suggests that some CD4+ CD161++ Vα7.2+ T cells may be conventional T cells expressing the Vα7.2 chain, consistent with a previous report (50). This heterogeneity of the CD4+ CD161++ Vα7.2+ T cell population can explain some of the differences observed between CD4+ and CD8+/DN CD161++ Vα7.2+ T cell populations, as the latter subsets mostly overlap with CD161++ MR1-tetramer+ cells. This shows that particularly when looking at CD4 populations, the MR1-tetramer should be used to accurately identify MAIT cells. Thus, when we compared the phenotypic differences between CD161++ Vα7.2+ T cell subsets with MR1-tetramer defined MAIT cell subsets, there was a significant difference between the level of marker expression in CD4+ CD161++ Vα7.2+ cells and CD4+ MR1-tetramer+ cells. Interestingly, however, CD4+ MR1-tetramer+ cells sorted using the MR1-tetramer were found to have significantly lower levels of Eomes and PLZF, and a significantly higher level of CCR4 compared to their CD4− counterparts, as found in CD161++ Vα7.2+ T cell subsets. The pattern of coexpression of these markers was also found to be similar between CD4+ MR1-tetramer+ cells and CD4+ CD161++ Vα7.2+ T cells. Thus, although there is less heterogeneity between MAIT cell subsets as defined by MR1-tetramers compared to CD161++ Vα7.2+ T cell subsets, the CD4 subset seems to be inherently more heterogeneous compared to the CD8/DN MAIT cell subset. Nevertheless, it will be important for future studies to further confirm any differences between MAIT cell subsets using the MR1-tetramer.

CD8+ and DN CD161++ Vα7.2+ T cells were functionally and phenotypically similar in this study and largely overlapped with 5-OP-RU-loaded MR1-tetramer stained cells. Thus, whether the CD8 coreceptor may affect the activation of CD8+ CD161++ Vα7.2+/MAIT cells was investigated. Although CD8α was not necessary for the activation of MAIT cells in our model, CD8+ MAIT cell responses were reduced in a dose-dependent manner by the addition of an anti-CD8α blocking antibody. As the structure and residues of the CD8-binding domain of MHC class I is conserved between MR1 and MHC class I molecules, it has been suggested that MR1 may bind CD8αα (4, 33). It is well attested that the binding of CD8 to MHC class I stabilizes the TCR/peptide–MHC class I interaction (51), and the results here support the idea that the CD8 coreceptor similarly stabilizes the MAIT TCR/MR1 interaction. We found that there was a hierarchy in cellular responses of MAIT cells that are affected by CD8 coreceptor blockade—the order was as follows, from least affected to most affected: MIP-1β < CD107a < TNFα ≤ IFNγ. This series is similar to the hierarchy in cellular responses elicited by peptide–MHC class I stimulation (52). Interestingly, studies using other activation models, such as *Helicobacter pylori*, have found a higher capacity of CD8+ MAIT cells to secrete cytokines and degranulate, suggesting that the CD8 coreceptor may play a more significant role in MR1-dependent activation in response to certain pathogens, or in response to low doses of antigen (53). Importantly, however, we cannot rule out that, in our assays, CD8 is acting as a simple adhesion molecule, or that the antibody is hindering the interaction between MR1 and the MAIT cell TCR. Furthermore, the CD8 coreceptor blockade may only be modifying the activation of CD8αβ+ MAIT cells. This is because the CD8β chain is required for efficient coreceptor function (54). A large fraction of the CD8+ MAIT cells express the CD8αα homodyne, which sequesters p56lck away from the TCR due to its exclusion from lipid rafts and, therefore, are thought to be inhibitory (55, 56). In order to confirm the function of the CD8 coreceptor on MAIT cell activation, further studies disrupting the potential CD8-binding site of MR1 will be necessary (57).

In conclusion, we have explored the heterogeneous as well as homogeneous phenotypes and functions of the three defined subsets of CD161++ Vα7.2+ T cells. Differences in TF expression, chemokine receptor expression, and capacity to secrete Th1 cytokines support the notion that different CD161++ Vα7.2+ T cell subsets, and the MAIT cells contained within this population, may play distinct roles in health and disease, and future studies will be warranted to further investigate the development and function of these cells.

## Ethics Statement

The study protocol conforms to the ethical guidelines of the 1975 Declaration of Helsinki as reflected in *a priori* approval by the institutions’ human research committees. Adult and cord blood samples were collected after ethical approval by the Central Office for Research Ethics Committees (COREC, local research ethics committee Oxford), reference number COREC 04.OXA.010. Liver samples were collected after ethic approval of the Asian American Liver Center Ethic committee (Glean Eagle Hospital, Singapore), reference number PIEC/2012/037. For samples used for gDNA analysis, samples were collected after ethical approval by the University of Otago Human Ethics Committee (Health), reference number H14/046. All participants from the studies above provided their informed written consent. The collection of blood samples for the 2-year-old study cohort was approved by the Human Ethics Committee at Huddinge University Hospital, Stockholm, reference code 75/97, 331/02, and the parents provided their informed verbal consent. No written documentation of the participants informed approval was required, which was agreed to by the Human Ethics Committee and was according to the regulations at the time of the initiation of the study.

## Author Contributions

AK designed and performed experiments, and wrote the manuscript. AJ, RH, JF, and LW performed experiments, and ES-E provided samples. AC provided the MR1-teramer and valuable advice, JU and CW provided advice and support, and PK supervised research work and data analysis.

## Conflict of Interest Statement

The authors declare that the research was conducted in the absence of any commercial or financial relationships that could be construed as a potential conflict of interest. The reviewer, EM, and handling editor declared their shared affiliation.

## Abbreviations

MAIT, mucosal-associated invariant T; DN, double-negative (CD4− CD8−); DP, double-positive (CD4+ CD8+); TCR, T cell receptor; MHC, major histocompatibility complex; MR1, MHC class I-related protein 1; Gr, Granzyme; *Escherichia coli, E. coli*; iNKT, CD1d-restricted invariant natural killer T cells; NK, natural killer cells; IHL, intrahepatic lymphocytes; BM, bone marrow; PLZF, promyelocytic leukemia zinc finger protein; RORγt, RAR-related orphan receptor γ t; 5-OP-RU, 5-(2-oxopropylideneamino)-6-d-ribitylaminouracil; 6-FP, 6-formyl pterin; β2M, β-2-microglobulin; Foxp3, forkhead box P3; gDNA, genomic DNA; ThPOK, Th-inducing POK; gMFI, geometric mean fluorescence intensity; T helper, Th; Eomes, eomesodermin; T-bet, T-box expressed in T cells; SEM, standard error of the mean; TF, transcription factor; PBS, phosphate buffered saline; PBMCs, peripheral blood mononuclear cells; FCS, fetal calf serum; PMA, phorbol 12-myristate 13-acetate; IL-4, interleukin-4; IFNγ, interferon-γ; TGF-β, transforming growth factor-β; MIP-1β, macrophage inflammatory protein-1β; TNFα, tumor necrosis factor-α; Yr., Year; FACS, fluorescence-activated cell sorted.
