# Supplementary material for: Shared and Distinct Phenotypes and Functions of Human CD161++ Vα7.2+ T Cell Subsets
Source: Front Immunol. 2017 Aug 30;8:1031. doi: 10.3389/fimmu.2017.01031 (PMC5582200; doi:10.3389/fimmu.2017.01031)
Supplement: Supplementary file 1 [file Table_1.PDF]

	CD161++Vα7.2+ T cells		
Marker	CD4	CD8	DN
CCR2	++	+++	+++
CCR4	+	-	-
CCR5	++	+++	+++
CCR6	+++	++	+++
CCR7	+	-	-
CD69	-	+	+
CD25	+	-	-
IL-18R	++	+++	+++
CD127	++	+++	+++
CD56	-	++	++
NKG2A	-	+	+
CD62L	+	-	-
RORγt	++	+++	+++
PLZF	++	+++	+++
Eomes	+	+++	+++
GrA	+	+++	+++
GrK	+	+++	+++
Perforin	-	+	+

Supplementary Table 1. Summary of markers that are significantly differentially expressed between CD4, CD8, and double-negative (DN) subsets of CD161++Vα7.2+T cell subsets. PLZF=promyelocytic leukaemia zinc finger, Eomes=Eomesdermin, GrA=Granzyme A.

+++ = high expression

++ = medium expression

+ = low expression

- = no expression
