# Supplementary material for: Shared and Distinct Phenotypes and Functions of Human CD161++ Vα7.2+ T Cell Subsets
Source: Front Immunol. 2017 Aug 30;8:1031. doi: 10.3389/fimmu.2017.01031 (PMC5582200; doi:10.3389/fimmu.2017.01031)
Supplement: Supplementary file 2 [file Image_1.PDF]

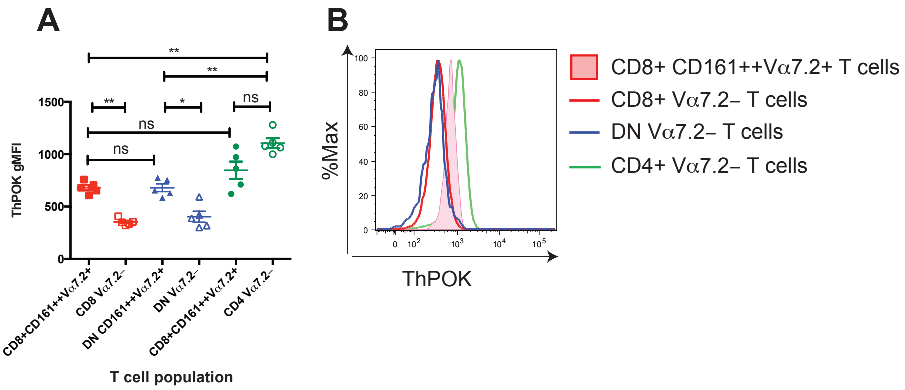

Supplementary Figure 1. Expression of ThPOK by blood CD161++Vα7.2+ T cell subsets.
A) geometric mean fluorescence intensity (gMFI) of ThPOK expressed in CD161++Vα7.2+ T cells (closed symbols) or conventional Vα7.2-negative T cells (open symbols) within CD8+, DN, or CD4+ T cells. **P<0.01, *P<0.05, ns=non-significant by repeated measures one-way ANOVA with Bonferroni's multiple comparisons test (n=5). Bars indicate mean ± S.E.M. B) Overlaid histogram showing the expression of ThPOK in CD8+ CD161++Vα7.2+ T cells (red tinted) compared to conventional Vα7.2- CD8+, DN, or CD4+ T cells. In all figures CD8+ cells are shown in red, DN cells in blue, and CD4+ cells in green.
