# Supplementary figures and images for: Shared and Distinct Phenotypes and Functions of Human CD161++ Vα7.2+ T Cell Subsets
Source: Front Immunol. 2017 Aug 30;8:1031. doi: 10.3389/fimmu.2017.01031 (PMC5582200; doi:10.3389/fimmu.2017.01031)

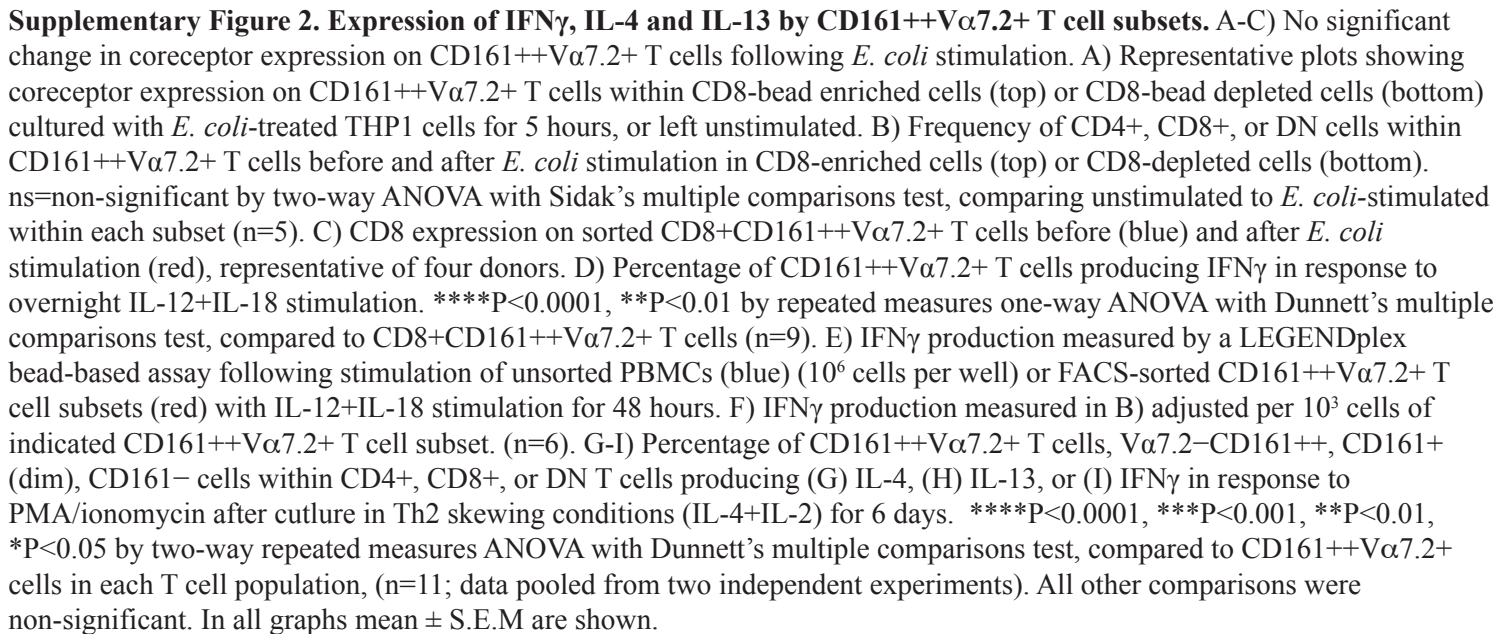

Supplement: Supplementary file 3 [file Image_2.PDF]
