# Supplementary material for: Shared and Distinct Phenotypes and Functions of Human CD161++ Vα7.2+ T Cell Subsets
Source: Front Immunol. 2017 Aug 30;8:1031. doi: 10.3389/fimmu.2017.01031 (PMC5582200; doi:10.3389/fimmu.2017.01031)
Supplement: Supplementary file 4 [file Image_3.PDF]

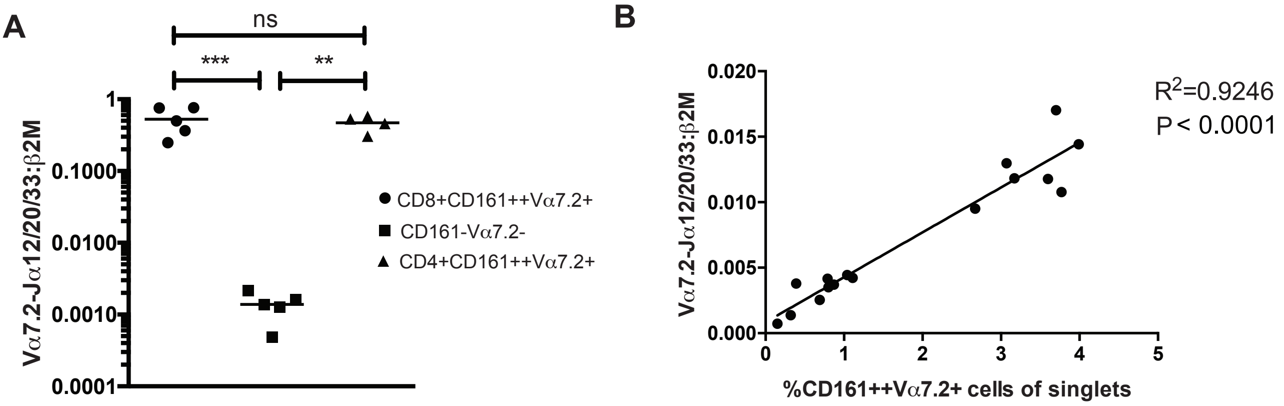

Supplementary Figure 3. Vα7.2-Jα33/20/12 genomic DNA (gDNA) rearrangement is enriched within CD4+ CD161++Vα7.2+ T cells. The abundance of Vα7.2-Jα33/20/12 genomic DNA (gDNA) rearangement in relation to β-2-microglobulin within FACS-sorted CD161++Vα7.2+ T cells was compared as previously described (Ussher et al., 2015). A) The relative abundance of Vα7.2-Jα33/20/12 gDNA in sorted CD8+ and CD4+ CD161++Vα7.2+ T cell subsets, compared to CD161−Vα7.2− T cells. ***P<0.001, **P<0.01, or ns=non-significant by one-way ANOVA with Tukey's multiple comparisons test. B) Pearson correlation analysis of the relative abundance of Vα 7.2-Jα33/20/12 gDNA relative to β-2-microglobulin (β2M) gDNA and the frequency of CD161++Vα7.2+ T cells among singlets. R^2 quantifies goodness of fit to the linear regression.
