# Supplementary material for: Shared and Distinct Phenotypes and Functions of Human CD161++ Vα7.2+ T Cell Subsets
Source: Front Immunol. 2017 Aug 30;8:1031. doi: 10.3389/fimmu.2017.01031 (PMC5582200; doi:10.3389/fimmu.2017.01031)
Supplement: Supplementary file 5 [file Image_4.PDF]

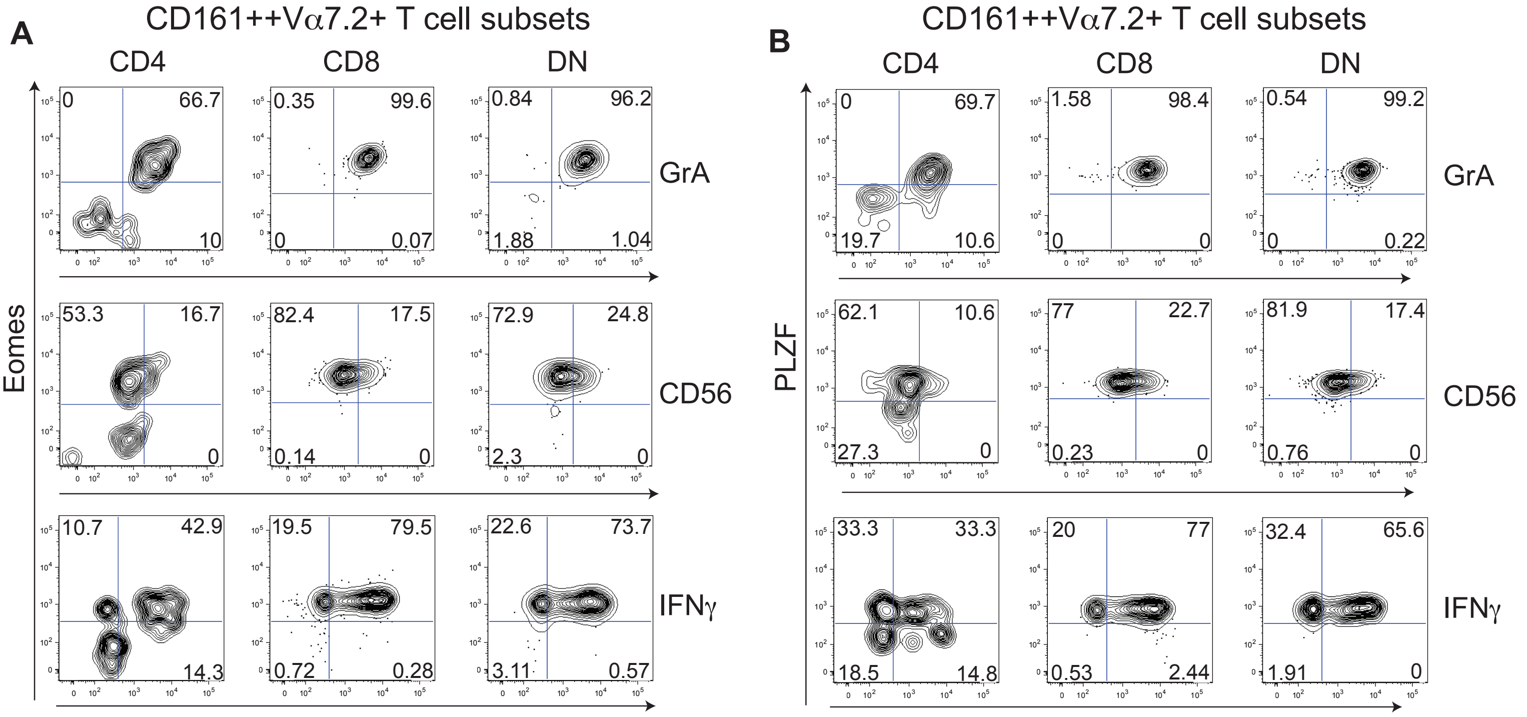

Supplementary Figure 4. Coexpression of Eomes and PLZF with Granzyme A, CD56, and IFNγ in CD161++Vα7.2+ T cell subsets. A-B) Coexpression of Eomes (A) or PLZF (B) with GrA (top), CD56 (middle), and IFNγ (bottom) in CD161++Vα7.2+ T cell subsets. For IFNγ expression, cells were stimulated with PMA/ionomycin for 5 hours and co-stained for transcription factors and IFNγ. Plots are representative of 10 donors.
