# Supplementary material for: Shared and Distinct Phenotypes and Functions of Human CD161++ Vα7.2+ T Cell Subsets
Source: Front Immunol. 2017 Aug 30;8:1031. doi: 10.3389/fimmu.2017.01031 (PMC5582200; doi:10.3389/fimmu.2017.01031)
Supplement: Supplementary file 6 [file Image_5.PDF]

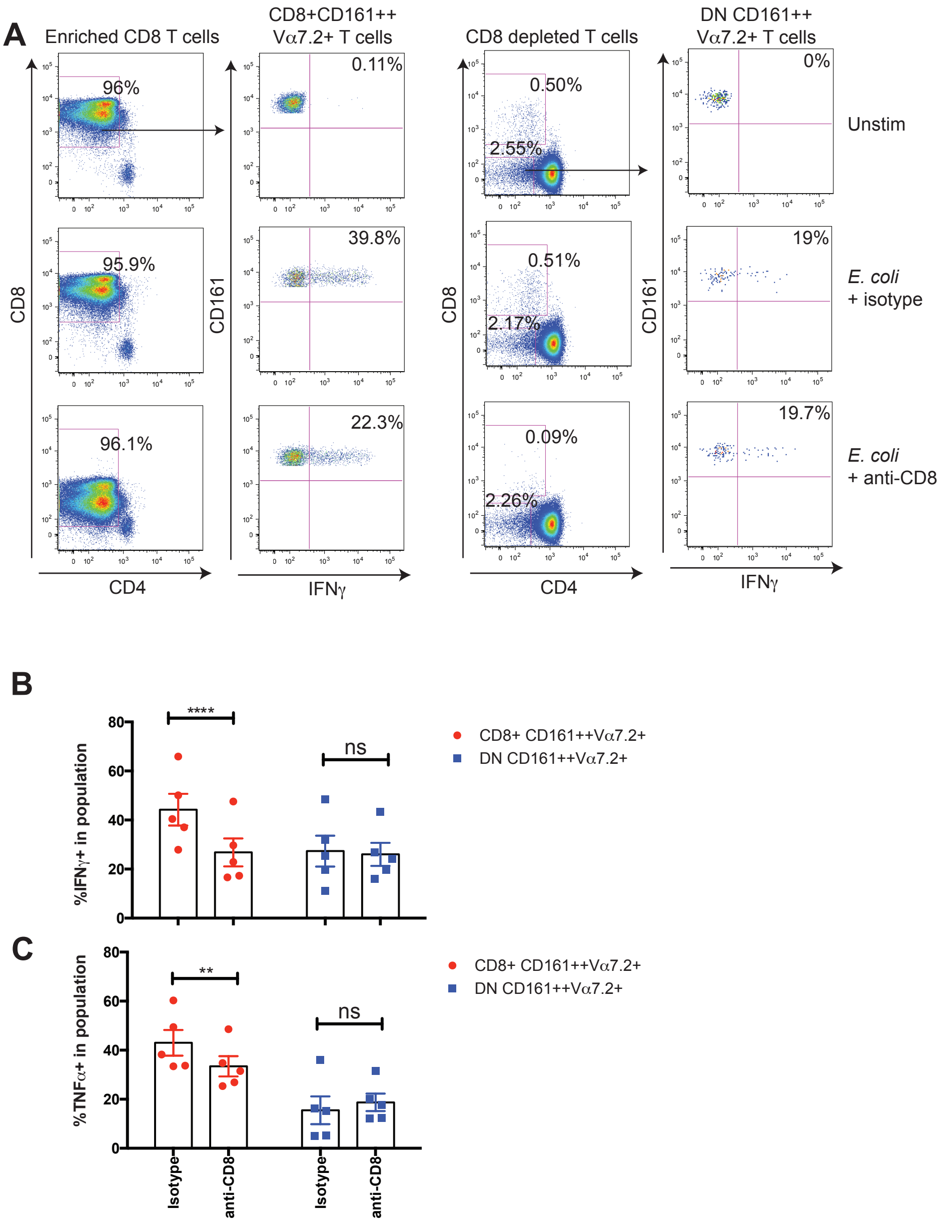

Supplementary Figure 5. Anti-CD8 blocking antibody does not affect the activation of DN CD161++Vα7.2+ T cells. A) Representative plots showing the effect of the anti-CD8 blocking antibody on IFNγ expression from either CD8+CD161++Vα7.2+ T cells within enriched CD8+ T cells (left) or DN CD161++Va7.2+ T cells within CD8-depleted T cells (right), both cultured with *E. coli*-treated THP1s for 5 hours. B-C) Data showing (B) IFNγ or (C) TNFα expression from either CD8+CD161++Vα7.2+ T cells within enriched CD8+ T cells or DN CD161++Vα7.2+T cells within CD8-depleted T cells, in the presence or absence of blocking antibody against CD8. ****P<0.0001, **P<0.01, ns=non-significant by two-way repeated measures ANOVA, with Bonferroni's multiple comparisons test, comparing the cells cultured with isotype controls versus cells cultured with anti-CD8 blocking antibody (n=5). Mean ± S.E.M are shown.
